# Pericolactines A–C, a New Class of Diterpenoid Alkaloids with Unusual Tetracyclic Skeleton

**DOI:** 10.1038/srep17082

**Published:** 2015-11-27

**Authors:** Yue-Hua Wu, Guo-Dong Chen, Rong-Rong He, Chuan-Xi Wang, Dan Hu, Gao-Qian Wang, Liang-Dong Guo, Xin-Sheng Yao, Hao Gao

**Affiliations:** 1 Institute of Traditional Chinese Medicine & Natural Products, College of Pharmacy, Jinan University, Guangzhou 510632, People’s Republic of China; 2 State Key Laboratory of Mycology, Institute of Microbiology, Chinese Academy of Sciences, Beijing 100190, People’s Republic of China

## Abstract

Fusicoccane diterpenoids usually possess a fused 5-8-5 tricyclic ring system, which are biogenetically generated from geranylgeranyl diphosphate (GGDP). In our report, three novel diterpenoid alkaloids with fusicoccane skeleton, pericolactines A–C (**1**–**3**), were isolated from *Periconia* sp.. Their structures with absolute configurations were determined by spectroscopic analyses and quantum chemical ECD calculation. Pericolactines A–C (**1**–**3**) are a new class of diterpenoid alkaloids with an unusual fused 5-5-8-5 tetracyclic ring system, which derive from a geranylgeranyl diphosphate (GGDP) and serine conjugated biosynthesis. They belong to the atypical diterpenoid alkaloids.

Diterpenoid alkaloids are a kind of nitrogen-containing diterpenes that derive from a terpene-amino acid conjugated biosynthesis. According their structural characteristics, the majority of them are classified as typical diterpenoid alkaloids, including C_18_-, C_19_-, and C_20_-diterpenoid alkaloids^1^,^2^,^3^,^4^. Till now, only a few atypical diterpenoid alkaloids have been reported, such as concavine, chamobtusin A, and haterumaimides^4^.

During our ongoing research on bioactive secondary metabolites from fungi^5^,^6^,^7^,^8^,^9^,^10^,^11^,^12^,^13^, a chemical investigation on metabolites from *Periconia* sp. (No. 19-4-2-1) isolated from the lichen *Parmelia* sp. was carried out, which led to the isolation of three novel polycyclic diterpenoid alkaloids, pericolactines A–C (**1**–**3**). Pericolactines A–C (**1**–**3**) are a new class of diterpenoid alkaloids featuring a fused 5-5-8-5 tetracyclic skeleton and belong to atypical diterpenoid alkaloids, which derive from the terpene-amino acid conjugated biosynthesis. Details of the structure elucidation for **1**–**3** (Fig. 1) are reported herein.

## Results

Pericolactine A (**1**), isolated as a white amorphous powder, was assigned the molecular formula C_24_H_35_NO_5_ (eight degrees of unsaturation) according to a quasi-molecular ion at *m/z* 418.2595 [M + H]^+^ in its HRESIMS spectrum. The ^13^C NMR spectrum (Table 1) showed 24 carbon signals, which was consistent with the deduction of the HRESIMS. Combined with the DEPT-135 experiment, these carbons can be categorized into six sp^2^ quaternary carbons [including two carbonyl carbons (*δ*_C_ 178.1 and 172.7) and four olefinic carbons], one sp^3^ quaternary carbon (*δ*_C_ 52.0), five sp^3^ methine carbons, eight sp^3^ methylene carbons, and four methyl carbons (*δ*_C_ 28.5, 20.8, 15.8, and 12.1, respectively). In the ^1^H NMR spectrum of **1** (Table 1), the characteristic protons for four methyl groups [*δ*_H_ 1.87 (s), 1.11 (s), 1.03 (d, *J* = 7.1 Hz), and 0.98 (d, *J* = 7.0 Hz)] were observed. All the proton resonances were assigned to relevant carbon atoms through the HSQC experiment. The analysis of the ^1^H-^1^H COSY experiment revealed the presence of five isolated spin systems (C-1–C-2–C-3(C-16)–C-4–C-5, C-8–C-9, C-12–C-13, C-19–C-15–C-20, and C-1′–C-2′) as shown in Fig. 2. Combined with the ^1^H-^1^H COSY analysis and the degrees of unsaturation, the HMBC correlations from Ha-1/Hb-1 to C-6, from H-2 to C-7, from H-4 to C-6, from H-5 to C-6/C-7/C-17, from Ha-8/Hb-8 to C-6/C-7/C-10/C-17, from Ha-9/Hb-9 to C-7/C-10/C-11, from Ha-12/Hb-12 to C-14, from Ha-13/Hb-13 to C-10, from H_3_-16 to C-2/C-3/C-4, from H_3_-18 to C-1/C-10/C-11/C-12 revealed a 5-8-5 fused ring system (rings A/B/C, Fig. 2). With the ^1^H-^1^H COSY correlations between Ha-19/Hb-19/H_3_-20 and H-15, the HMBC correlations from H-15 to C-10/C-13/C-14, from Ha-19/Hb-19 to C-14/C-15/C-20/19-OCOCH_3_, H_3_-20 to C-14/C-15/C-19, and from 19-OCOCH_3_ to 19-OCOCH_3_ revealed a 1-acetoxypropan-2-yl located at C-14. With the ^1^H-^1^H COSY correlations between Ha-1′/Hb-1′ and Ha-2′/Hb-2′, the key HMBC correlations from Ha-1′/Hb-1′ to C-5/C-17 revealed a γ-lactam ring in **1** (ring D, Fig. 2). On the basis of the analyses of ^1^H-^1^H COSY, HMBC, the degrees of unsaturation, and the molecular formula, the planar structure of **1** was deduced as shown in Fig. 2, and the assignments of all proton and carbon resonances are shown in Table 1.

The ROESY correlations between H-2 and H-4/H-5, and between H-3 and H-5 signified that H-2, H-3, H-4, and H-5 located on the same face of the ring A (Fig. 3). Furthermore, the ROESY correlation between H-2 and Ha-12 signified that H-2 and C-12 located on the same face of the ring B (Fig. 3), while H_3_-18 was on the other face. Combined with the ROESY correlations between H-5 and 19-OCOCH_3_, between Ha-9 and H-15, between Hb-13 and Ha-19/Hb-19, and between Ha-13/Hb-13 and H_3_-20, the relative configurations of C-2, C-3, C-4, C-5, C-11, and C-15 in **1** were assigned as 2S*, 3R*, 4R*, 5S*, 11R*, and 15R*, respectively (Fig. 3). Thus, the structure of **1** was established as a new diterpenoid alkaloids with fusicoccane skeleton.

Pericolactine B (**2**) was obtained as a white amorphous powder. It was assigned the molecular formula C_22_H_33_NO_4_ (seven degrees of unsaturation) according to a quasi-molecular ion at *m/z* 376.2491 [M + H]^+^ in its HRESIMS spectrum. The molecular weight of **2** was a 42 atomic mass unit (C_2_H_2_O) less than **1**, which indicated that **2** may be a 19-deacetylated derivative of **1**. The ^1^H and ^13^C NMR spectra of **2** showed resonances very similar to **1**, except for the disappearance of one acetyl group. Further detailed NMR analyses involving ^1^H-^1^H COSY and HMBC spectra (see Supplementary Table S2 online) confirmed the above deduction and given the assignments of all proton and carbon resonances (Table 1).

The fact that **2** is the deacetylated derivative of **1** was confirmed by acid hydrolysis. Pericolactine A (**1**) was treated with H_2_SO_4_ in MeOH, and then the product prepared from **1** was compared with **2** using HPLC (see Supplementary Figure S1 online), which displayed that the retention times of the product prepared from **1** were identical to **2** isolated from fungal broth in three eluting systems. Based on the above mentioned fact, the relative configuration of **2** was assigned as 2S*, 3R*, 4R*, 5S*, 11R*, 15R*, which was the same as **1**. The absolute configurations of C-2, C-3, C-4, C-5, C-11, and C-15 in **2** were determined by quantum chemical ECD calculation. The conformational analysis for a pair of enantiomers ((2*S*, 3*R*, 4*R*, 5*S*, 11*R*, 15*R*)-**2** and (2*R*, 3*S*, 4*S*, 5*R*, 11*S*, 15*S*)-**2**) was carried out in CONFLEX version 7.0 with an energy window for acceptable conformers (0**–**3 kcal mol^−1^). The acceptable conformers were obtained, and continued to be optimized in Gaussian09. After that, five lowest energy conformers were found out. These lowest energy conformers (Fig. 4) were submitted to the ECD calculation at [B3P86/6-311++G (2d, p)] level, and the predicted ECD curve of (2*S*, 3*R*, 4*R*, 5*S*, 11*R*, 15*R*)-**2** was similar to the experimental one (Fig. 5 and see Supplementary information). Therefore, the absolute configuration of **2** was established as 2*S*, 3*R*, 4*R*, 5*S*, 11*R*, and 15*R*.

**Figure 1:**
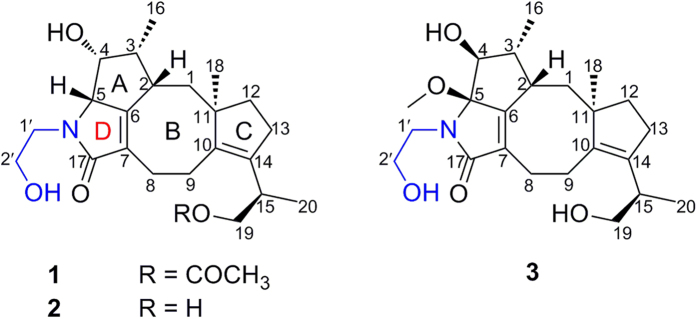
Chemical structures of 1–3.

**Figure 2:**
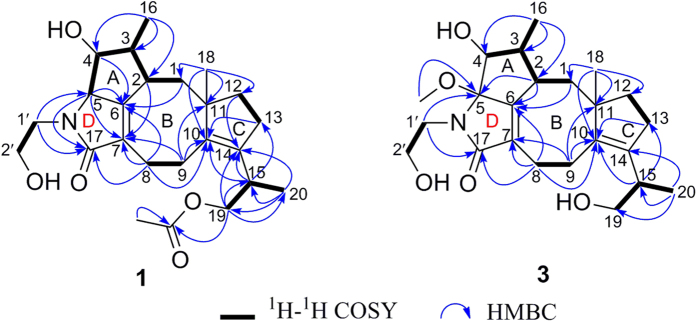
Key ^1^H-^1^H COSY and HMBC correlations of 1 and 3.

**Figure 3:**
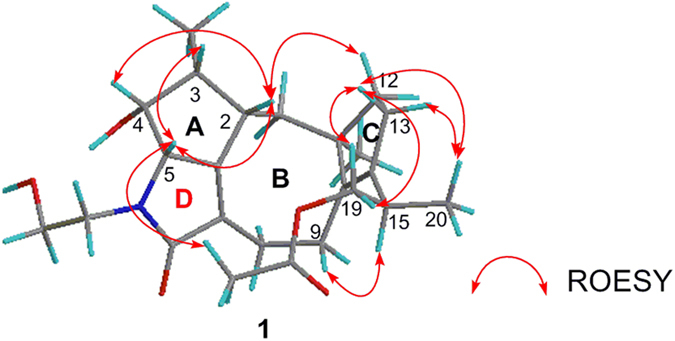
Key ROESY correlations of 1.

**Figure 4:**
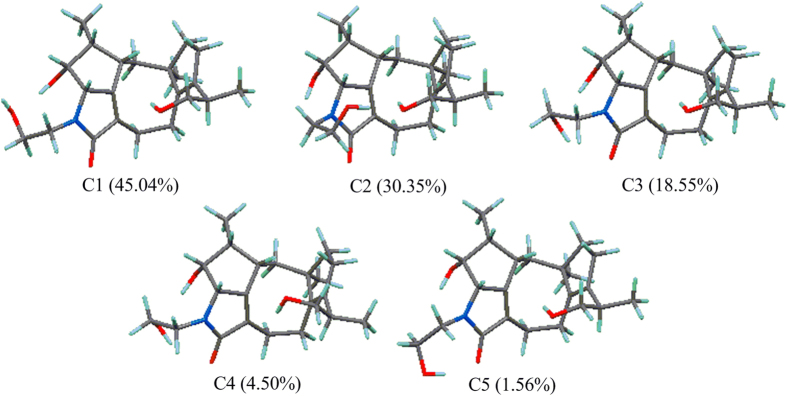
Most stable conformers of (2*S*, 3*R*, 4*R*, 5*S*, 11*R*, 15*R*)-2 (the relative populations are in parentheses).

**Figure 5:**
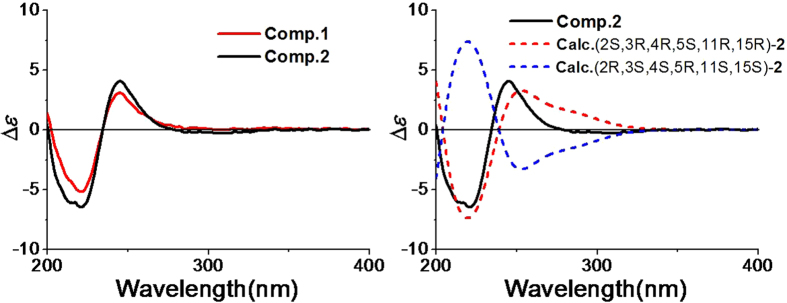
Experimental ECD spectra of 1–2 and calculated ECD spectra of (2*S*, 3*R*, 4*R*, 5*S*, 11*R*, 15*R*)-2 and (2*R*, 3*S*, 4*S*, 5*R*, 11*S*, 15S)-2.

Since **1** and **2** possess the similar ECD curves (Fig. 5) and **1** and **2** coexist in the same strain, **1** and **2** possess the same absolute configurations. Thus, the absolute configuration of **1** was also assigned as 2*S*, 3*R*, 4*R*, 5*S*, 11*R*, and 15*R*.

Pericolactine C (**3**) was isolated as a white amorphous powder. Its molecular formula was established as C_23_H_35_NO_5_ (seven degrees of unsaturation) by the quasi-molecular ion at *m/z* 428.2418 [M + Na]^+^ in the HRESIMS. The ^1^H and ^13^C NMR spectra of **3** were very similar to **2**, expect for the absence of C-5 methine and the appearance of an oxygenated sp^3^ quaternary carbon (*δ*_C_ 97.0) and a methoxy group (*δ*_C_ 50.6/*δ*_H_ 2.99). The key HMBC correlation from the additional methoxy group at *δ*_H_ 2.99 to C-5 (*δ*_C_ 97.0) indicated that H-5 in **2** was substituted by methoxy group in **3**. On the basis of 2D NMR analysis (see Supplementary Table S3 online), the planar structure of **3** was established (Fig. 2), and the assignments of all proton and carbon resonances are shown in Table 1.

**Figure 6:**
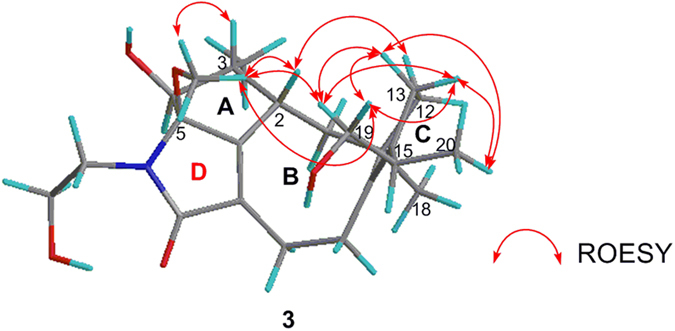
Key ROESY correlations of 3.

**Figure 7:**
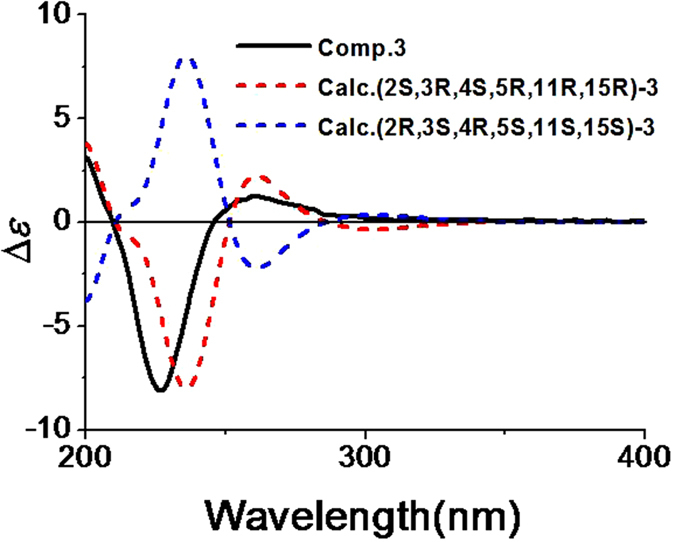
Experimental ECD spectra of 3 and calculated ECD spectra of (2*S*, 3*R*, 4*S*, 5*R*, 11*R*, 15*R*)-3 and (2*R*, 3*S*, 4*R*, 5*S*, 11*S*, 15*S*)-3.

The relative configuration of **3** was elucidated by analysis of the ROESY experiment. The ROESY correlations between 5-OCH_3_ and H-2/H-3 signified that H-2, H-3, and 5-OCH_3_ located on the same face of the ring A (Fig. 6). Furthermore, the ROESY correlation between H-2 and Ha-12 signified that H-2 and C-12 located on the same face of the ring B (Fig. 6), while the H_3_-18 was on the other face. Combined with the ROESY correlations between 5-OCH_3_ and Ha-19/Hb-19, and between Ha-19/Hb-19/H_3_-20 and Ha-13/Hb-13, the relative configurations of C-2, C-3, C-5, C-11, and C-15 were assigned as 2S*, 3R*, 5R*, 11R*, and 15R* (Fig. 6). However, the coupling constant between H-3 and H-4 (^3^*J*_H-3, H-4_ = 8.8 Hz) of **3** was different from **1** and **2** (^3^*J*_H-3, H-4_ = 3.7 Hz), which suggested that the epimerization was at C-4 in **3**. Therefore, the relative configuration of **3** was determined as 2S*, 3R*, 4S*, 5R*, 11R*, and 15R*. The absolute configuration of **3** was determined by quantum chemical ECD calculation. The conformational analysis for a pair of enantiomers ((2*S*,3*R*,4*S*,5*R*,11*R*,15*R*)-**3** and (2*R*,3*S*,4*R*,5*S*,11*S*,15*S*)-**3**) was carried out in CONFLEX version 7.0 with an energy window for acceptable conformers (0**–**3 kcal mol^−1^). The acceptable conformers were obtained, and continued to be optimized in Gaussian09. After that, only one lowest energy conformer was found out. The lowest energy conformer was submitted to the ECD calculation at [B3P86/6-311++G (2d, p)] level, and the predicted ECD curve of (2*S*, 3*R*, 4*S*, 5*R*, 11*R*, 15*R*)-**3** was similar to the experimental one (Fig. 7 and see Supplementary information). On the basis of the above analyses, the absolute configuration of **3** was assigned as 2*S*, 3*R*, 4*S*, 5*R*, 11*R*, and 15*R*.

All isolated compounds were subjected to a paper disk-diffusion assay^14^,^15^ for antimicrobial activities against two bacteria (*Staphylococcus aureus* 209P and *Escherichia coli* ATCC0111) and two fungi (*Candida albicans* FIM709 and *Aspergillus niger* R330). In addition, all isolated compounds were also evaluated by MTT method^16^,^17^ for their cytotoxicity against five human tumor cell lines, including HL-60, SMMC-7721, A-549, MCF-7, and SW480, with cisplatin and paclitaxel as the positive controls. However, compounds showed no potent activity (see Supplementary Table S4 and S5 online).

## Discussion

Fusicoccane diterpenoids usually possessing a tricyclic (5-8-5) ring system (such as brassicicenes, cyclooctatins, fusicoccins, and periconicins) are biogenetically generated from geranylgeranyl diphosphate (GGDP)^18^, which are found from various natural sources, including bacteria^19^,^20^, fungi^21^,^22^,^23^,^24^, liverworts^25^,^26^, algas^27^, and higher plants^28^,^29^. Fusicoccane diterpenoids exhibit diverse biological activities, such as plant growth regulating activity (fusicoccins)^30^, lysophospholipase inhibitory activity (cyclooctatin)^31^, antimicrobial activity (periconicins)^32^,^33^, nitrification inhibitory activity (brachialactone)^34^, cytotoxicity against tumor cells (cotylenins)^35^, inhibiting insulin-stimulated GLUT4 fusion activity (fusicoccins)^35^, and so on.

**Figure 8:**
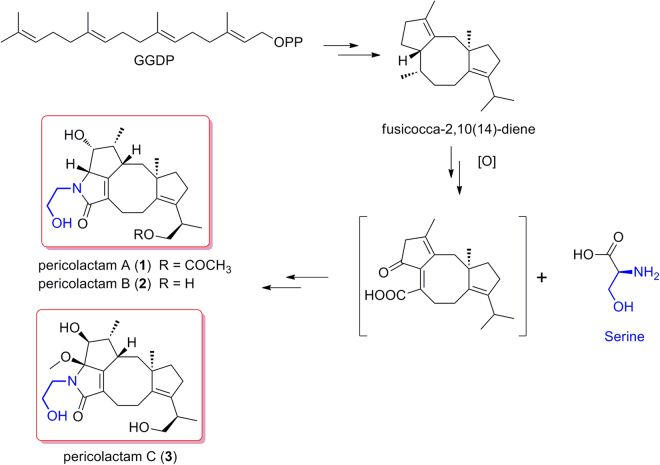
Plausible biogenetic pathway of 1–3.

Pericolactines A–C (**1**–**3**) are the first nitrogen-containing fusicoccane diterpenoids, which derive from fusicocca-2,10(14)-diene and serine (Figure 8). Due to the participation of serine in the biogenetic pathway, pericolactines A–C (**1**–**3**) represent a new class of diterpenoid alkaloids.

## Materials and Methods

### General experimental procedures

Optical rotations were measured on a JASCO P1020 digital polarimeter, and UV data were obtained with a JASCO V-550 UV/vis spectrometer. The CD spectra were recorded in MeOH using a JASCO J-810 spectrophotometer at room temperature. IR data were recorded using JASCO FT/IR-480 Plus spectrometer. HRESIMS spectra were obtained on Waters Synapt G2 TOF mass spectrometer. The NMR data were acquired with a Bruker AV 400 NMR spectrometer using solvent signals (CD_3_OD: *δ*_H_ 3.30/*δ*_C_ 49.0) as standards. Column chromatography (CC) was carried out on Sephadex LH-20 (Pharmacia, USA), and ODS (60–80 μm, YMC). TLC was performed on precoated silica gel plate (SGF254, 0.2 mm, Yantai Chemical Industry Research Institute, China). Analytical HPLC was performed on a Dionex HPLC system equipped with an Ultimate 3000 pump, an Ultimate 3000 diode array detector, an Ultimate 3000 column compartment, an Ultimate 3000 autosampler (Dionex, USA), and an Alltech (Grace) 2000ES evaporative light scattering detector (Alltech USA) using a Phenomenex Gemini C18 column (4.6 × 250 mm, 5 μm). Preparative HPLC was carried out on Shimadzu LC-6AD system equipped with UV detectors, using a Phenomenex Gemini C18 column (21.2 × 250 mm, 5 μm). Semi-preparative HPLC was carried out on Shimadzu LC-6AD system equipped with UV detectors, using a YMC-Pack ODS-A column (10.0 × 250 mm, 5 μm).

### Fungus material

The strain of *Periconia* sp. (No. 19-4-2-1) was isolated by one of the authors (L.D. Guo) from the lichen *Parmelia* sp. collected from Changbai Mountain, Jilin Province, China, in August 2006. The fungus strain was identified as *Periconia* sp. based on the morphological characteristics and sequence analysis of the internal transcribed spacer (ITS) regions ITS1-5.8S-ITS2 (GenBank accession No. KP873157). Briefly, the genomic DNA of the fungus was extracted and used as a template for amplification of ITS region by fungus-specific universal primer pair ITS1 (5′-TCCGTAGGTGAACCTGCGG-3′) and ITS4 (5′-TCCTCCGCTTATTGATATGC-3′). The resulting DNA fragment was sequenced and deposited at GenBank. Species were identified by searching databases using the BLAST sequence analysis tool (http://www.ncbi.nlm.nih.gov/BLAST/). The strain was assigned the accession number 19-4-2-1 in the culture collection at the Institute of Traditional Chinese Medicine and Natural Products, college of Pharmacy, Jinan University, Guangzhou. The fungus was cultured on slants of potato dextrose agar at 25 °C for 5 days. Agar plugs were used to inoculate four Erlenmeyer flasks (250 mL), each containing 100 mL of potato dextrose broth. Four flasks of the inoculated media were incubated at 25 °C on a rotary shaker at 200 rpm for 5 days to prepare the seed culture. Fermentation was carried out in 20 Erlenmeyer flasks (500 mL), each containing 70 g of rice. Distilled H_2_O (105 mL) was added to each flask, and the rice was soaked overnight before autoclaving at 120 °C for 30 min. After cooling to room temperature, each flask was inoculated with 5.0 mL of the spore inoculum and incubated at room temperature for 45 days.

### Extraction and isolation

The culture was extracted thrice with EtOAc, and the organic solvent was evaporated to dryness under vacuum to afford a crude extract (33.4 g). The crude extract was dissolved in 90% v/v aqueous MeOH (500 mL) and partitioned against the same volume of cyclohexane to afford a cyclohexane fraction (C, 24.5 g) and an aqueous MeOH fraction (W, 8.7 g). The aqueous MeOH fraction (W, 8.7 g) was separated by ODS CC eluting with MeOH-H_2_O (30:70, 50:50, 70:30, and 100:0, v/v) to afford four fractions (W1 to W4). Fraction W3 (2.6 g) was further separated on a ODS column with a gradient of MeOH-H_2_O (55:45, 60:40, 65:35, 70:30, and 100:0, v/v) to give seven subfractions (W3a to W3 g). Subfraction W3c (1.3 g) was subjected to Sephadex LH-20 CC using MeOH to afford four portions (W3c1 to W3c4). W3c3 (994.5 mg) was separated on preparative HPLC using CH_3_CN-H_2_O (35:65, v/v) to yield **1** (6.8 mg) and **3** (4.4 mg). Fraction W2 (2.3 g) was also subjected to a ODS column with a gradient of MeOH-H_2_O (35:65, 40:60, 45:55, 50:50, 55:45, and 100:0, v/v) to give eight subfractions (W2a to W2h). Subfraction W2f (399.6 mg) was separated by ODS CC eluting with MeOH-H_2_O (50:50, v/v) to afford three portions (W2f1 to W2f3). W2f2 (299.0 mg) was purified on semi-preparative HPLC by using CH_3_CN-H_2_O (35: 65, v/v) to yield **2** (11.2 mg).

### Spectroscopic data of 1–3

Pericolactine A (**1**): white amorphous powder; 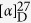 −29.1 (*c* 1.0, MeOH); UV (MeOH) *λ*_max_ (log *ε*) 207 (3.97) nm; CD (*c* 2.7 × 10^−4^ M, MeOH) *λ*_max_ (Δ*ε*) 220 (−5.17), 245 (+3.10); IR (KBr) *ν*_max_ 3437, 2944, 1718, 1658, 1385, 1240, 1038 cm^−1^; HRESI-TOF-MS *m/z* 418.2595 [M + H]^+^ (calcd for C_24_H_36_NO_5_, 418.2593); The ^1^H and ^13^C NMR data, see Supplementary Table S1 online.

Pericolactine B (**2**): white amorphous powder; 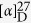 −33.5 (*c* 1.0, MeOH); UV (MeOH) *λ*_max_ (log *ε*) 209 (3.86) nm; CD (*c* 3.4 × 10^−4^ M, MeOH) *λ*_max_ (Δ*ε*) 220 (−6.48), 245 (+4.10); IR (KBr) *ν*_max_ 3390, 2941, 1656, 1034 cm^−1^; HRESI-TOF-MS *m/z* 376.2491 [M + H]^+^ (calcd for C_22_H_34_NO_4_, 376.2488); The ^1^H and ^13^C NMR data, see Supplementary Table S2 online.

Pericolactine C (**3**): white amorphous powder; 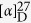 −27.6 (*c* 1.0, MeOH); UV (MeOH) *λ*_max_ (log *ε*) 207 (4.04) nm; CD (*c* 3.0 × 10^−4^ M, MeOH) *λ*_max_ (Δ*ε*) 226 (−8.09), 259 (+1.29); IR (KBr) *ν*_max_ 3403, 2941, 1681, 1385, 1050 cm^−1^; HRESI-TOF-MS *m/z* 428.2418 [M + Na]^+^ (calcd for C_23_H_35_NO_5_Na, 428.2413); The ^1^H and ^13^C NMR data, see Supplementary Table S3 online.

### Acid hydrolysis of 1

Compound **1** (1.0 mg) stirred with 98% H_2_SO_4_ (2 μL) in MeOH (2 mL) at 40 °C for 3.5 h. After neutralization with ammonia, the solvent was evaporated to yield the mixture. Then the mixture was compared with **2** by HPLC, which displayed that the retention time of the product prepared from compound **1** was identical to **2** isolated from fungal broth (see Supplementary Figure S1 online).

## Additional Information

**How to cite this article**: Wu, Y.-H. *et al.* Pericolactines A–C, a New Class of Diterpenoid Alkaloids with Unusual Tetracyclic Skeleton. *Sci. Rep.* **5**, 17082; doi: 10.1038/srep17082 (2015).

## Supplementary Material

Supplementary Information

## Figures and Tables

**Table 1:** ^1^H (400 MHz) and ^13^C (100 MHz) NMR data of **1**–**3** in CD_3_OD (*δ* in ppm, *J* in Hz).

No.	1		2		3	
	*δ*_C_	*δ_H_	*δ*_C_	*δ_H_	*δ*_C_	*δ_H_
1	41.2	1.68, dd (14.1, 1.0), a	41.5	1.67, dd (14.0, 1.4), a	39.8	1.77, dd (14.1, 12.7), a
		1.57, dd (14.0, 11.0), b		1.59, dd (13.9, 11.1), b		1.63, dd (14.0, 2.0), b
2	37.0	2.70, br t (11.4)	37.1	2.76, br t (11.0)	36.8	2.90, br t (12.6)
3	45.1	2.80, dqd (11.0, 7.0, 3.6)	44.9	2.81, dqd (11.0, 7.1, 3.6)	48.3	2.63
4	73.4	4.04, t (3.7)	73.4	4.02, t (3.7)	83.2	3.25, d (8.8)
5	72.1	4.27	71.7	4.26	97.0	
6	166.7		167.3		161.3	
7	129.1		128.6		132.0	
8	22.9	2.60, a	22.1	2.58, a	21.9	2.60
		2.48, b		2.51, b		
9	23.7	2.48, a	24.1	2.48, a	23.7	2.47, a
		2.13, b		2.15, b		2.20, b
10	142.1		141.1		140.1	
11	52.0		51.9		51.6	
12	37.6	1.78, a	38.6	1.78, a	37.5	1.83, a
		1.71, b		1.71, b		1.69, b
13	30.0	2.31, a	30.4	2.27, a	30.2	2.30, a
		2.23, b		2.18, b		2.20, b
14	140.0		141.5		142.2	
15	33.6	2.89, br sext (7.1)	37.0	2.67	37.2	2.60
16	12.1	1.03, d (7.1)	12.1	1.01, d (7.0)	15.0	1.02, d (7.2)
17	178.1		178.1		174.4	
18	28.5	1.11, s	28.6	1.14, s	28.9	1.14, s
19	68.7	3.93, dd (10.6, 7.7), a	66.9	3.34, dd (10.0, 7.5), a	66.8	3.37, dd (10.5, 6.2), a
		3.86, dd (10.6, 6.6), b		3.33, dd (10.0, 6.3), b		3.30, b
20	15.8	0.98, d (7.0)	15.7	0.95, d (6.9)	16.0	0.94, d (6.9)
19-OCOCH_3_	172.7					
19-OCOCH_3_	20.8	1.87, s				
5-OCH_3_					50.6	2.99, s
1′	45.9	3.62, a	45.9	3.62, a	42.3	3.64, a
		3.40, b		3.39, b		3.16, b
2′	61.3	3.74, a	61.3	3.75, a	61.8	3.69
		3.70, b		3.68, b		

^*^The indiscernible signals due to overlap or have the complex multiplicity are reported without designating multiplicity.

## References

[b1] Pelletier S. W. & Page S. W. Diterpenoid alkaloids. Nat Prod Rep 1, 375–386 (1984). 10.1039/np9860300451 3547189

[b2] Pelletier S. W. & Page S. W. Diterpenoid alkaloids. Nat Prod Rep 3, 451–464 (1986). 3547189 10.1039/np9860300451

[b3] Atta-ur-Rahman & Choudhary M. I. Diterpenoid and steroidal alkaloids. Nat Prod Rep 16, 619–635 (1999). 10584334 10.1039/a705715f

[b4] Wang F. P., Chen Q. H. & Lu X. Y. Diterpenoid alkaloids. Nat Prod Rep 27, 529–570 (2010). 20336236 10.1039/b916679c

[b5] Chen G. D. *et al.* Xanthoquinodins from the endolichenic fungal strain *Chaetomium elatum*. J Nat Prod 76, 702–709 (2013). 23586920 10.1021/np400041y

[b6] Ye F. *et al.* Xinshengin, the first altenusin with tetracyclic skeleton core from *Phialophora* spp. Tetrahedron Lett 54, 4551–4554 (2013).

[b7] Zheng Q. C. *et al.* Nodulisporisteriods A and B, the first 3,4-*seco*-4-methyl-progesteroids from *Nodulisporium* sp. Steroids 78, 896–901 (2013). 23685090 10.1016/j.steroids.2013.05.007

[b8] Wu Z. Y. *et al.* Xylariterpenoids A-D, four new sesquiterpenoids from the Xylariaceae fungus. RSC Adv 4, 54144–54148 (2014).

[b9] Xiong H. *et al.* Sporormiellin A, the first tetrahydrofuran-fused furochromone with an unprecedented tetracyclic skeleton from *Sporormiella minima*. RSC Adv 4, 24295–24299 (2014).

[b10] Zhao Q. *et al.* Nodulisporiviridins A-H, bioactive viridins from *Nodulisporium* sp.. J Nat Prod 78, 1221–1230 (2015). 25978520 10.1021/np500912t

[b11] Zhao Q. *et al.* Nodulisporisteroids C-L, new 4-methyl-progesteroid derivatives from *Nodulisporium* sp. steroids 102, 101–109 (2015). 26254609 10.1016/j.steroids.2015.08.004

[b12] Bao Y. R. *et al.* 4,5-*seco*-Probotryenols A-C, a new type of sesquiterpenoids from *Stachybotrys bisbyi*. RSC Adv 5, 46252–46259 (2015).

[b13] Wu Y. H. *et al.* Pericoterpenoid A, a new bioactive cadinane-type sesquiterpene from *Periconia* sp.. J Asian Nat Prod Res 17, 671–675 (2015). 26096303 10.1080/10286020.2015.1049162

[b14] Groblacher B., Maier V., Kunert O. & Bucar F. J Nat Prod 75, 1393–1399 (2012). 22789014 10.1021/np300375t

[b15] Shen C. C., Syu W. J., Li S. Y., Lin C. H., Lee G. H. & Sun C. M. J Nat Prod 65, 1857–1862 (2002). 12502328 10.1021/np010599w

[b16] Mosmman T. J Immunol Methods 65, 55–63 (1983).6606682

[b17] Alley M. C., Scudiero D. A., Monks A., Hursey M. L., Czerwinski M. J. & Fine D. L. Cancer Res 48, 589–601 (1988). 3335022

[b18] Arens J., Engels B., Klopries S., Jennewein S., Ottmann C. & Schulz F. Exploration of biosynthetic access to the shared precursor of the fusicoccane diterpenoid family. Chem Commun 49, 4337–4339 (2013). 10.1039/c2cc37154e 23295536

[b19] Aoyama T., Naganawa H., Muraoka Y., Aoyagi T. & Takeuchi T. The structure of cyclooctatin, a new inhibitor of lysophospholipase. J Antibiot 45, 1703–1704 (1992). 1474002 10.7164/antibiotics.45.1703

[b20] Kawamura A., Iacovidou M., Hirokawa E., Soll C. E. & Trujillo M. 17-Hydroxycyclooctatin, a fused 5-8-5 ring diterpene, from *Streptomyces* sp. MTE4a. J Nat Prod 74, 492–495 (2011). 21314175 10.1021/np100921m PMC3064735

[b21] Billio A., Chain E. B., de Leo P., Erlanger B. F., Mauri M. & Tonolo A. Fusicoccin: a new wilting toxin produced by *Fusicoccum amygdali* Del. Nature 203, 297 (1964).

[b22] Sassa T. Cotylenins, leaf growth substances produced by a fungus Part I. isolation and characterization of cotylenins A and B. Agric Biol Chem 35, 1415–1418 (1971).

[b23] MacKinnon S. L., Keifer P. & Ayer W. A. Components from the phytotoxic extract of *Alternaria brassicicola*, a black spot pathogen of canola. Phytochemistry 51, 215–221 (1999).

[b24] Takekawa H., Tanaka K., Fukushi E., Matsuo K. & Nehira T. Roussoellols A and B, tetracyclic fusicoccanes from *Roussoella hysterioides*. J Nat Prod 76, 1047–1051 (2013). 23692046 10.1021/np400045z

[b25] Hashimoto T., Tori M., Taira Z. & Asakawa Y. New highly oxidized fusicoccane diterpenoids from the liverwort *Plagiochila acathophylla* subsp. *Japonica*. Tetrahedron Lett 26, 6473–6476 (1985).

[b26] Liu N., Guo D. X., Wang S. Q., Wang Y. Y., Zhang L., Li G. & Lou H. X. Bioactive sesquiterpenoids and diterpenoids from the liverwort *Bazzania albifolia*. Chem Biodivers 9, 2254–2261 (2012). 23081925 10.1002/cbdv.201100408

[b27] Enoki N., Furusaki A., Suehiro K., Ishida R. & Matsumoto T. Epoxydictymene, a new diterpene from the brown alga *Dictyota dichotoma*. Tetrahedron Lett 24, 4341–4342 (1983).

[b28] Okogun J. I., Adesomoju A. A., Adesida G. A., Lindner H. J. & Habermehl G. Roseanolone: a new diterpene from *Hypoestes rosea*. Z Naturforsch Sec C 37c, 558–561 (1982)

[b29] Adesomoju A. A., Okogun J. I., Cava M. P. & Carroll P. J. Roseadione, a diterpene ketone from *Hypoestes rosea*. Phytochemsitry 22, 2535–2536 (1983).

[b30] Marre E. Fusicoccin: a tool in plant physiology. Annu Rev Plant Physiol 30, 273–288 (1979).

[b31] Kim S. Y. *et al.* Cloning and heterologous expression of the cyclooctatin biosynthetic gene cluster afford a diterpene cyclase and two P450 hydroxylases. Chem Biol 16, 736–743 (2009). 19635410 10.1016/j.chembiol.2009.06.007

[b32] Kim S., Shin D. S., Lee T. & Oh K. B. Periconicins, two new fusicoccane diterpenes produced by an endophytic fungus *Periconia* sp. with antibacterial activity. J Nat Prod 67, 448–450 (2004). 15043428 10.1021/np030384h

[b33] Shin D. S., Oh M. N., Yang H. C. & Oh K. B. Biological characterization of periconicins, bioactive secondary metabolites, produced by *Periconia* sp. OBW-15. J Microbiol Biotechnol 15, 216–220 (2005).

[b34] Subbarao G. V. *et al.* Evidence for biological nitrification inhibition in *Brachiaria* pastures. Proc Natl Acad Sci U.S.A. 106, 17302–17307 (2009). 19805171 10.1073/pnas.0903694106 PMC2752401

[b35] de Boer A. H. & Leeuwen I. J. D. Fusicoccanes: diterpenes with surprising biological functions. Trends Plant Sci 17, 360–368 (2012). 22465041 10.1016/j.tplants.2012.02.007

