# Supplementary material for: Pericolactines A–C, a New Class of Diterpenoid Alkaloids with Unusual Tetracyclic Skeleton
Source: Sci Rep. 2015 Nov 27;5:17082. doi: 10.1038/srep17082 (PMC4661464; doi:10.1038/srep17082)
Supplement: Supplementary Information [file srep17082-s1.doc]

**SUPPLEMENTARY INFORMATION**

**Pericolactines A−C, a New Class of Diterpenoid Alkaloids with Unusual Tetracyclic Skeleton**

Yue-Hua Wu,^1,+^ Guo-Dong Chen,^1,+^ Rong-Rong He,^1,*^Chuan-Xi Wang,^1^ Dan Hu,^1^ Gao-Qian Wang,^1^ Liang-Dong Guo,^2^ Xin-Sheng Yao,^1^ and Hao Gao^1,*^

^1^Institute of Traditional Chinese Medicine & Natural Products, College of Pharmacy, Jinan University, Guangzhou 510632, People’s Republic of China

^2^State Key Laboratory of Mycology, Institute of Microbiology, Chinese Academy of Sciences, Beijing 100190, People’s Republic of China

^*^Corresponding author: tghao@jnu.edu.cn (Hao Gao); rongronghe66@163.com (Rong-Rong He)

^+^These authors contributed equally to this work.

List of Supporting Information

[1. The NMR data assignments of 1−3 2](#__RefHeading___Toc429583653)

[2. Acid hydrolysis of 1 5](#__RefHeading___Toc429583654)

[3. Quantum chemical ECD calculation of 2 and 3 6](#__RefHeading___Toc429583655)

[4. Antimicrobial assay and Cytotoxicity assay of 1−3 19](#__RefHeading___Toc429583656)

[5. The spectra of 1−3 21](#__RefHeading___Toc429583657)

1. The NMR data assignments of 1−3

Table S1 ^13^C NMR (100 MHz) and ^1^H NMR (400 MHz) data of **1** in CD_3_OD

| **position** | ***δ*_C_, mult.** | ****δ*_H_ (*J* in Hz)** | **^1^H-^1^H COSY** | **HMBC** | **ROESY** |
| --- | --- | --- | --- | --- | --- |
| 1 | 41.2, CH_2_ | 1.68, dd (14.1, 1.0), a | 1b, 2 | 2, 3, 6, 10, 11, 12, 18 | 16, 18 |
|  |  | 1.57, dd (14.0, 11.0), b | 1a, 2 | 6, 11, 12, 18 | 9b, 16, 18 |
| 2 | 37.0, CH | 2.70, br t (11.4) | 1a, 1b, 3 | 1, 3, 4, 6, 7, 11 | 4, 5, 12a, 13b |
| 3 | 45.1, CH | 2.80, dqd (11.0, 7.0, 3.6) | 2, 4, 16 | 2, 16 | 5 |
| 4 | 73.4, CH | 4.04, t (3.7) | 3, 5 | 2, 6 | 2, 16, 1'b, 2'a, 2'b |
| 5 | 72.1, CH | 4.27 | 4, 8a, 8b | 6, 7 | 2, 3, 1'b, 19-OCOCH_3_ |
| 6 | 166.7, C |  |  |  |  |
| 7 | 129.1, C |  |  |  |  |
| 8 | 22.9, CH_2_ | 2.60, a | 5, 8b, 9a | 6, 7, 9, 10, 17 |  |
|  |  | 2.48, b | 5, 8a, 9b | 6, 7, 9, 10, 17 |  |
| 9 | 23.7, CH_2_ | 2.48, a | 8a, 9b | 7, 8, 10, 11, 14 | 15 |
|  |  | 2.13, b | 8b, 9a | 7, 8, 10, 11, 14 | 1b, 18 |
| 10 | 142.1, C |  |  |  |  |
| 11 | 52.0, C |  |  |  |  |
| 12 | 37.6, CH_2_ | 1.78, a | 12b, 13a, 13b | 1, 10, 11, 13, 14, 18 | 2 |
|  |  | 1.71, b | 12a, 13a, 13b | 1, 10, 11, 13, 14, 18 |  |
| 13 | 30.0, CH_2_ | 2.31, a | 12a, 12b, 13b | 10, 12, 14 | 18, 20 |
|  |  | 2.23, b | 12a, 12b, 13a | 10, 12, 14 | 2, 19a, 19b, 20 |
| 14 | 140.0, C |  |  |  |  |
| 15 | 33.6, CH | 2.89, br sext (7.1) | 19a, 19b, 20 | 10, 13, 14, 19, 20 | 9a |
| 16 | 12.1, CH_3_ | 1.03, d (7.1) | 3 | 2, 3, 4 | 1a, 1b, 4 |
| 17 | 178.1, C |  |  |  |  |
| 18 | 28.5, CH_3_ | 1.11, s |  | 1, 10, 11, 12 | 1a, 1b, 9b, 13a |
| 19 | 68.7, CH_2_ | 3.93, dd (10.6, 7.7), a | 15, 19b | 14, 15, 20, 19-OCOCH_3_ | 13b |
|  |  | 3.86, dd (10.6, 6.6), b | 15, 19a | 14, 15, 20, 19-OCOCH_3_ | 13b |
| 20 | 15.8, CH_3_ | 0.98, d (7.0) | 15 | 14, 15, 19 | 13a, 13b |
| 19-OCOCH_3_ | 172.7, C |  |  |  |  |
| 19-OCOCH_3_ | 20.8, CH_3_ | 1.87, s |  | 19-OCOCH_3_ | 5 |
| 1′ | 45.9, CH_2_ | 3.62, a | 1′b, 2′a, 2′b | 5, 17, 2' |  |
|  |  | 3.40, b | 1′a, 2′a, 2′b | 5, 17, 2' | 4, 5 |
| 2′ | 61.3, CH_2_ | 3.74, a | 1′a, 1′b, 2′b | 1' | 4 |
|  |  | 3.70, b | 1′a, 1′b, 2′a | 1' | 4 |

* The indiscernible signals due to overlap or have the complex multiplicity are reported without designating multiplicity

Table S2 ^13^C NMR (100 MHz) and ^1^H NMR (400 MHz) data of **2** in CD_3_OD

| **position** | ***δ*_C_, mult.** | ****δ*_H_ (*J* in Hz)** | **^1^H-^1^H COSY** | **HMBC** | **ROESY** |
| --- | --- | --- | --- | --- | --- |
| 1 | 41.5, CH_2_ | 1.67, dd (14.0, 1.4), a | 1b, 2 | 2, 3, 6, 10, 11, 12, 18 | 8a, 16, 18 |
|  |  | 1.59, dd (13.9, 11.1), b | 1a, 2 | 2, 6, 10, 11, 12, 18 | 9b, 16, 18 |
| 2 | 37.1, CH | 2.76, br t (11.0) | 1a, 1b, 3 | 1, 3, 4, 6 | 4, 5, 12a, 16 |
| 3 | 44.9, CH | 2.81, dqd (11.0, 7.1, 3.6) | 2, 4, 16 | 1, 2, 16 | 5 |
| 4 | 73.4, CH | 4.02, t (3.7) | 3, 5 | 2, 6 | 2, 16, 2′a, 2′b |
| 5 | 71.7, CH | 4.26 | 4, 8a, 8b | 6, 7, 17 | 2, 3, 1′b, 2′a, 2′b |
| 6 | 167.3, C |  |  |  |  |
| 7 | 128.6, C |  |  |  |  |
| 8 | 22.1, CH_2_ | 2.58, a | 5, 8b, 9a, 9b | 6, 7, 9, 10, 17 | 1a |
|  |  | 2.51, b | 5, 8a, 9a, 9b | 6, 7, 9, 10, 17 |  |
| 9 | 24.1, CH_2_ | 2.48, a | 8a, 8b, 9b | 7, 8, 10, 11, 14 |  |
|  |  | 2.15, b | 8a, 8b, 9a | 7, 8, 10, 14 | 1b, 18 |
| 10 | 141.1, C |  |  |  |  |
| 11 | 51.9, C |  |  |  |  |
| 12 | 38.6, CH_2_ | 1.78, a | 12b, 13a, 13b | 1, 10, 11, 13, 14, 18 | 2 |
|  |  | 1.71, b | 12a, 13a, 13b | 1, 10, 11, 13, 14, 18 |  |
| 13 | 30.4, CH_2_ | 2.27, a | 12a, 12b, 13b | 10, 12, 14 | 19a, 19b, 20 |
|  |  | 2.18, b | 12a, 12b, 13a | 10, 12, 14 | 20 |
| 14 | 141.5, C |  |  |  |  |
| 15 | 37.0, CH | 2.67 | 19a, 19b, 20 | 10, 13, 14, 19, 20 |  |
| 16 | 12.1, CH_3_ | 1.01, d (7.0) | 3 | 2, 3, 4 | 1a, 1b, 2, 4 |
| 17 | 178.1, C |  |  |  |  |
| 18 | 28.6, CH_3_ | 1.14, s |  | 1, 10, 11, 12 | 1a, 1b, 9b |
| 19 | 66.9, CH_2_ | 3.34, dd (10.0, 7.5), a | 15, 19b | 14, 15, 20 | 13a |
|  |  | 3.33, dd (10.0, 6.3), b | 15, 19a | 14, 15, 20 | 13a |
| 20 | 15.7, CH_3_ | 0.95, d (6.9) | H-15 | 14, 15, 19 | 13a, 13b |
| 1′ | 45.9, CH_2_ | 3.62, a | 1′b, 2′a, 2′b | 5, 17, 2′ |  |
|  |  | 3.39, b | 1′a, 2′a, 2′b | 5, 17, 2′ | 5 |
| 2′ | 61.3, CH_2_ | 3.75, a | 1′a, 1′b, 2′b | 1′ | 4, 5 |
|  |  | 3.68, b | 1′a, 1′b, 2′a | 1′ | 4, 5 |

* The indiscernible signals due to overlap or have the complex multiplicity are reported without designating multiplicity

Table S3 ^13^C NMR (100 MHz) and ^1^H NMR (400 MHz) data of **3** in CD_3_OD

| **position** | ***δ*_C_, mult.** | ****δ*_H_ (*J* in Hz)** | **^1^H-^1^H COSY** | **HMBC** | **ROESY** |
| --- | --- | --- | --- | --- | --- |
| 1 | 39.8, CH_2_ | 1.77, dd (14.1, 12.7), a | 1b, 2 | 2, 6, 11, 12, 18 | 8, 16, 18 |
|  |  | 1.63, dd (14.0, 2.0), b | 1a, 2 | 2, 3, 6, 10, 11, 12 | 16, 18 |
| 2 | 36.8, CH | 2.90, br t (12.6) | 1a, 1b, 3 | 5, 6 | 12a, 13b, 16, 5-OCH_3_ |
| 3 | 48.3, CH | 2.63 | 2, 4, 16 | 4, 6 | 5-OCH_3_ |
| 4 | 83.2, CH | 3.25, d (8.8) | 3 | 3, 16 | 16 |
| 5 | 97.0, C |  |  |  |  |
| 6 | 161.3, C |  |  |  |  |
| 7 | 132.0, C |  |  |  |  |
| 8 | 21.9, CH_2_ | 2.60 | 9a, 9b | 6, 7, 9, 10 | 1a |
| 9 | 23.7, CH_2_ | 2.47, a | 8, 9b | 7, 8, 10, 11, 14 |  |
|  |  | 2.20, b | 8, 9a | 7, 8, 10, 11, 14 | 18 |
| 10 | 140.1, C |  |  |  |  |
| 11 | 51.6, C |  |  |  |  |
| 12 | 37.5, CH_2_ | 1.83, a | 12b, 13a, 13b | 1, 11, 13, 14, 18 | 2 |
|  |  | 1.69, b | 12a, 13a, 13b | 1, 10, 11, 13, 14, 18 | 18 |
| 13 | 30.2, CH_2_ | 2.30, a | 12a, 12b, 13b | 10, 12, 14 | 19a, 19b, 20 |
|  |  | 2.20, b | 12a, 12b, 13a | 10, 12, 14 | 2, 19a, 19b, 20 |
| 14 | 142.2, C |  |  |  |  |
| 15 | 37.2, CH | 2.60 | 19a, 19b, 20 | 10, 13, 14, 19, 20 |  |
| 16 | 15.0, CH_3_ | 1.02, d (7.2) | 3 | 2, 3, 4 | 1a, 1b, 2, 4 |
| 17 | 174.4, C |  |  |  |  |
| 18 | 28.9, CH_3_ | 1.14, s |  | 1, 10, 11, 12 | 1a, 1b, 9b, 12b |
| 19 | 66.8, CH_2_ | 3.37, dd (10.5, 6.2), a | 15, 19b | 14, 15, 20 | 13a, 13b, 5-OCH_3_ |
|  |  | 3.30, b | 15, 19a | 14, 15, 20 | 13a, 13b, 5-OCH_3_ |
| 20 | 16.0, CH_3_ | 0.94, d (6.9) | 15 | 14, 15, 19 | 13a, 13b |
| 5-OCH_3_ | 50.6, CH_3_ | 2.99, s |  | 5 | 2, 3, 19a, 19b, 1′b, 2′ |
| 1′ | 42.3, CH_2_ | 3.64, a | 1′b, 2′ | 5, 17, 2′ |  |
|  |  | 3.16, b | 1′a, 2′ | 5, 17, 2′ | 5-OCH_3_ |
| 2′ | 61.8, CH_2_ | 3.69 | 1'a, 1′b | 1′ | 5-OCH_3_ |

* The indiscernible signals due to overlap or have the complex multiplicity are reported without designating multiplicity

2. Acid hydrolysis of 1

Compound **1** (1.0 mg) stirred with 98% H_2_SO_4_ (2 μL) in MeOH (2 mL) at 40 °C for 3.5 h. After neutralization with ammonia, the solvent was evaporated to yield the mixture. Then the mixture was compared with **2** by HPLC, which displayed that the retention time of the product prepared from compound **1** was identical to **2** isolated from fungal broth.

Figure S1 The product prepared from **1** was compared with **2** by HPLC with three eluting systems (a: MeOH–H_2_O (70:30, v/v), at a flow rate of 1 mL/min; b: MeOH–H_2_O (60:40, v/v), at a flow rate of 1 mL/min; c: MeCN–H_2_O (35:65, v/v), at a flow rate of 1 mL/min)

3. Quantum chemical ECD calculation of 2 and 3

**Quantum chemical ECD calculation of 2**

The molecules of (2*S*,3*R*,4*R*,5*S*,11*R*,15*R*)-**2** and (2*R*,3*S*,4*S*,5*R*,11*S*,15*S*)-**2** were converted into SMILES codes before their initial 3D structures were generated with CORINA version 3.4. Conformer databases were generated in CONFLEX version 7.0 using the MMFF94s force-field, with an energy window for acceptable conformers (ewindow) of 3 kcal mol^-1^ above the ground state, a maximum number of conformations per molecule (maxconfs) of 100, and an RMSD cutoff (rmsd) of 0.5Å. Then each conformer of the acceptable conformers was optimized with HF/6-31G(d) method in Gaussian09^1^. Further optimization at the B3P86/6-31G(d) level led the dihedral angles to be got. After that, five lowest energy conformers were found out. The optimized conformers were taken for the ECD calculations, which were performed with Gaussian09 (B3P86/6-311++G(2d,p)). The solvent effects were taken into account by the polarizable-conductor calculation model (CPCM, methanol as the solvent). Comparisons of the experimental and calculated spectra were done with the software SpecDis^2,3^. It was also used to apply a UV shift to the ECD spectra, Gaussian broadening of the excitations, and Boltzmann weighting of the spectra.

Table S4 Conformers distribution of (2*S*,3*R*,4*R*,5*S*,11*R*,15*R*)-**2** in solvated models calculations at the B3P86/6-31G (d)

| Conformers | Contribution % |
| --- | --- |
| 1 | 45.04 |
| 2 | 30.35 |
| 3 | 18.55 |
| 4 | 4.50 |
| 5 | 1.56 |

Figure S2 Experimental ECD spectrum of **2** and calculated ECD spectra for (2*S*,3*R*,4*R*,5*S*,11*R*,15*R*)-**2** and (2*R*,3*S*,4*S*,5*R*,11*S*,15*S*)-**2** (UV correction = 0 nm, band width σ = 0.4 eV)

**Quantum chemical ECD calculation of 3**

The molecules of (2*S*,3*R*,4*S*,5*R*,11*R*,15*R*)-**3** and (2*R*,3*S*,4*R*,5*S*,11*S*,15*S*)-**3** were converted into SMILES codes before their initial 3D structures were generated in CORINA version 3.4. Conformer databases were generated in CONFLEX version 7.0 using the MMFF94s force-field, with an energy window for acceptable conformers (ewindow) of 3 kcal mol^-1^ above the ground state, a maximum number of conformations per molecule (maxconfs) of 100, and an RMSD cutoff (rmsd) of 0.5Å. Then each conformer of the acceptable conformers was optimized with HF/6-31G(d) method in Gaussian09^1^. Further optimization at the B3P86/6-31G(d) level led the dihedral angles to be got. After that, one lowest energy conformer was found out. The optimized conformer was taken for the ECD calculations, which was performed with Gaussian09 (B3P86/6-311++G(2d,p)). The solvent effects were taken into account by the polarizable-conductor calculation model (CPCM, methanol as the solvent). Comparisons of the experimental and calculated spectra were done with the software SpecDis^2,3^. It was also used to apply a UV shift to the ECD spectra, and Gaussian broadening of the excitations.

Figure S3 Experimental ECD spectrum of **3** and calculated ECD spectra for (2*S*,3*R*,4*S*,5*R*,11*R*,15*R*)-**3** and (2*R*,3*S*,4*R*,5*S*,11*S*,15*S*)-**3** (UV correction = 0 nm, band width σ = 0.3 eV)

**References**

1. Frisch, M.J., *et al*. Gaussian, Inc., Wallingford CT, **2010**.
2. Bruhn, T., Schaumlöffel, A., Hemberger, Y. & Bringmann, G.; Version 1.61 ed.; University of Würzburg: Würzburg, Germany, **2013**.
3. Bruhn, T., Schaumlöffel, A., Hemberger, Y. & Bringmann, G. SpecDics: quantifying the comparison of calculated and experimental electronic circular dichroism spectra. *Chirality* **25**, 243–249 (2013).

**Coordinates of computation** (2*S*,3*R*,4*R*,5*S*,11*R*,15*R*)-**2**

C1

Standard orientation:

----------------------------------------------------------------------------------------------------

Center Atomic Atomic Coordinates (Angstroms)

Number Number Type X Y Z

-----------------------------------------------------------------------------------------------------

1 6 0 0.597259 0.067368 -0.171056

2 6 0 0.630882 -0.670505 0.954794

3 6 0 -0.229237 -0.659534 2.176711

4 6 0 -1.738781 -0.841308 1.930461

5 6 0 -2.339743 0.039247 0.872681

6 6 0 -2.188261 1.562353 0.901306

7 6 0 -0.759707 2.064938 0.550124

8 6 0 -0.092765 1.325802 -0.612995

9 6 0 1.063713 2.063052 -1.400603

10 6 0 2.360473 1.279018 -1.105653

11 6 0 1.829568 -0.172311 -0.989123

12 7 0 2.568524 -1.131926 -0.213711

13 6 0 1.876298 -1.492170 0.913097

14 6 0 -3.099981 -0.380217 -0.160397

15 6 0 -3.562672 0.774156 -1.017415

16 6 0 -3.204578 2.014003 -0.186999

17 6 0 -3.465831 -1.798685 -0.522321

18 6 0 -4.936170 -1.945770 -0.925170

19 6 0 -2.558015 -2.322699 -1.644603

20 8 0 -1.206461 -2.448045 -1.248972

21 8 0 2.255473 -2.320680 1.739819

22 6 0 3.806757 -1.769157 -0.599600

23 6 0 5.004149 -1.241049 0.181818

24 8 0 5.220769 0.156768 0.003962

25 1 0 -0.871318 1.084367 -1.348861

26 1 0 1.608234 -0.561191 -1.994368

27 8 0 2.921757 1.712794 0.105910

28 6 0 1.203112 3.559222 -1.170014

29 6 0 -2.569588 2.147211 2.267135

30 1 0 -0.057984 0.274227 2.726905

31 1 0 0.118923 -1.462549 2.836045

32 1 0 -2.247207 -0.660481 2.888241

33 1 0 -1.920473 -1.890869 1.681176

34 1 0 -0.106383 2.017582 1.428556

35 1 0 -0.855461 3.129326 0.307588

36 1 0 0.859057 1.906978 -2.467417

37 1 0 3.077838 1.384774 -1.933530

38 1 0 -3.061111 0.766343 -1.996894

39 1 0 -4.636253 0.724437 -1.233862

40 1 0 -2.810318 2.832314 -0.798619

41 1 0 -4.107397 2.394515 0.303968

42 1 0 -3.289805 -2.448739 0.342914

43 1 0 -5.190919 -3.003268 -1.053394

44 1 0 -5.151219 -1.435576 -1.870753

45 1 0 -5.599725 -1.528597 -0.160813

46 1 0 -2.884821 -3.326664 -1.937810

47 1 0 -2.657595 -1.675165 -2.531964

48 1 0 -0.945281 -1.610415 -0.826829

49 1 0 3.727711 -2.848112 -0.419990

50 1 0 3.952657 -1.620492 -1.674689

51 1 0 4.825843 -1.382089 1.250050

52 1 0 5.903411 -1.805762 -0.094157

53 1 0 5.566850 0.300682 -0.889966

54 1 0 3.750390 1.195535 0.216890

55 1 0 0.288235 4.091164 -1.451529

56 1 0 2.019668 3.959289 -1.781563

57 1 0 1.427995 3.783817 -0.124393

58 1 0 -1.878777 1.829763 3.056680

59 1 0 -3.580747 1.840659 2.557706

60 1 0 -2.547282 3.243200 2.231855

-----------------------------------------------------------------------------------------------------

C2

Standard orientation:

--------------------------------------------------------------------------------------------------------

Center Atomic Atomic Coordinates (Angstroms)

Number Number Type X Y Z

--------------------------------------------------------------------------------------------------------

1 6 0 0.678391 0.607870 0.033794

2 6 0 0.738772 0.167107 1.305822

3 6 0 -0.250180 0.266944 2.419339

4 6 0 -1.617194 -0.379678 2.105352

5 6 0 -2.198879 -0.026953 0.766777

6 6 0 -2.493511 1.424047 0.370038

7 6 0 -1.223685 2.276593 0.084098

8 6 0 -0.168005 1.555931 -0.751819

9 6 0 0.935408 2.406540 -1.510190

10 6 0 2.305918 1.873242 -1.023153

11 6 0 1.987540 0.405097 -0.655123

12 7 0 2.814431 -0.233080 0.343653

13 6 0 2.074557 -0.466023 1.493339

14 6 0 -2.548907 -0.903290 -0.198888

15 6 0 -3.125684 -0.201426 -1.405899

16 6 0 -3.329778 1.243708 -0.929202

17 6 0 -2.406324 -2.407841 -0.184288

18 6 0 -3.670336 -3.121838 -0.675362

19 6 0 -1.200560 -2.858928 -1.019227

20 8 0 0.032347 -2.434593 -0.446545

21 8 0 2.478564 -1.079101 2.476072

22 6 0 3.922940 -1.104881 -0.013832

23 6 0 3.474383 -2.517273 -0.391803

24 8 0 2.611137 -2.542181 -1.512022

25 1 0 -0.694669 0.981783 -1.526277

26 1 0 1.922494 -0.211845 -1.558921

27 8 0 2.707232 2.583297 0.129978

28 6 0 0.815995 3.919502 -1.398720

29 6 0 -3.313020 2.150203 1.443890

30 1 0 -0.395282 1.318872 2.690377

31 1 0 0.177343 -0.221053 3.301889

32 1 0 -2.313503 -0.081156 2.900774

33 1 0 -1.509253 -1.465443 2.185243

34 1 0 -0.775518 2.626985 1.020526

35 1 0 -1.561748 3.177390 -0.440885

36 1 0 0.866794 2.142820 -2.572324

37 1 0 3.068767 1.952957 -1.811200

38 1 0 -2.444149 -0.267749 -2.267251

39 1 0 -4.066130 -0.657296 -1.736820

40 1 0 -3.061613 1.981465 -1.692700

41 1 0 -4.388558 1.402418 -0.696180

42 1 0 -2.210222 -2.742889 0.841187

43 1 0 -3.579464 -4.202761 -0.525332

44 1 0 -3.847247 -2.947259 -1.742250

45 1 0 -4.552881 -2.776628 -0.127761

46 1 0 -1.159521 -3.951798 -1.052568

47 1 0 -1.296120 -2.495339 -2.053129

48 1 0 -0.067122 -1.496855 -0.193624

49 1 0 4.459652 -0.641939 -0.847063

50 1 0 4.606663 -1.158898 0.839250

51 1 0 4.369191 -3.104773 -0.634612

52 1 0 3.000338 -2.985638 0.480391

53 1 0 1.685638 -2.561389 -1.183820

54 1 0 3.306292 1.991958 0.616851

55 1 0 -0.124475 4.274216 -1.831947

56 1 0 1.633980 4.403942 -1.943437

57 1 0 0.865413 4.250014 -0.358308

58 1 0 -2.748378 2.275199 2.375066

59 1 0 -4.232409 1.600800 1.675418

60 1 0 -3.596605 3.150148 1.094344

--------------------------------------------------------------------------------------------------------

C3

Standard orientation:

---------------------------------------------------------------------------------------------------------

Center Atomic Atomic Coordinates (Angstroms)

Number Number Type X Y Z

---------------------------------------------------------------------------------------------------------

1 6 0 0.590189 0.073919 -0.268737

2 6 0 0.680421 -0.615337 0.886995

3 6 0 -0.124788 -0.548905 2.144309

4 6 0 -1.639724 -0.756891 1.961280

5 6 0 -2.285705 0.067638 0.885325

6 6 0 -2.154224 1.591839 0.843132

7 6 0 -0.740034 2.099142 0.449536

8 6 0 -0.084907 1.343111 -0.707377

9 6 0 1.092401 2.065374 -1.483956

10 6 0 2.367044 1.252912 -1.171911

11 6 0 1.795616 -0.189379 -1.121109

12 7 0 2.524286 -1.180211 -0.384260

13 6 0 1.947371 -1.394144 0.837978

14 6 0 -3.075599 -0.407257 -0.100170

15 6 0 -3.581118 0.700590 -0.993298

16 6 0 -3.197074 1.984088 -0.243603

17 6 0 -3.440260 -1.844415 -0.380556

18 6 0 -4.928447 -2.024790 -0.695595

19 6 0 -2.587271 -2.406497 -1.526560

20 8 0 -1.214287 -2.494128 -1.200293

21 8 0 2.461406 -2.056167 1.745697

22 6 0 3.784820 -1.781985 -0.748857

23 6 0 4.981309 -0.915005 -0.347126

24 8 0 4.772957 -0.304408 0.922166

25 1 0 -0.861963 1.114620 -1.449159

26 1 0 1.560258 -0.528758 -2.138553

27 8 0 2.876917 1.648877 0.077128

28 6 0 1.257231 3.557867 -1.246742

29 6 0 -2.519709 2.226368 2.191403

30 1 0 0.057027 0.411848 2.641615

31 1 0 0.256897 -1.313926 2.829279

32 1 0 -2.116002 -0.536776 2.927188

33 1 0 -1.817647 -1.818272 1.765833

34 1 0 -0.068252 2.084103 1.314633

35 1 0 -0.853310 3.155322 0.179880

36 1 0 0.899264 1.915572 -2.553832

37 1 0 3.119397 1.368499 -1.966954

38 1 0 -3.122738 0.645045 -1.992067

39 1 0 -4.662683 0.634782 -1.160282

40 1 0 -2.816596 2.762999 -0.912808

41 1 0 -4.085050 2.398533 0.246625

42 1 0 -3.209035 -2.454171 0.501040

43 1 0 -5.175364 -3.089511 -0.766618

44 1 0 -5.201647 -1.556252 -1.647611

45 1 0 -5.554708 -1.584087 0.086815

46 1 0 -2.913075 -3.427331 -1.755558

47 1 0 -2.745666 -1.801582 -2.435018

48 1 0 -0.948695 -1.636612 -0.823442

49 1 0 3.835592 -2.739766 -0.222213

50 1 0 3.803331 -1.985851 -1.823944

51 1 0 5.891464 -1.528872 -0.347306

52 1 0 5.133560 -0.094522 -1.055158

53 1 0 4.319789 -0.956448 1.491250

54 1 0 3.530780 0.985413 0.392822

55 1 0 0.357345 4.108503 -1.539777

56 1 0 2.089497 3.942504 -1.847024

57 1 0 1.471778 3.776074 -0.197629

58 1 0 -1.812062 1.947058 2.980456

59 1 0 -3.522191 1.920214 2.510845

60 1 0 -2.510481 3.320278 2.112761

---------------------------------------------------------------------------------------------------------

C4

Standard orientation:

-------------------------------------------------------------------------------------------------------------

Center Atomic Atomic Coordinates (Angstroms)

Number Number Type X Y Z

-------------------------------------------------------------------------------------------------------------

1 6 0 0.575287 0.033988 -0.221341

2 6 0 0.687481 -0.621830 0.951332

3 6 0 -0.099966 -0.515084 2.217102

4 6 0 -1.617252 -0.723466 2.055802

5 6 0 -2.266598 0.058349 0.952256

6 6 0 -2.173785 1.582120 0.876553

7 6 0 -0.769895 2.081384 0.433990

8 6 0 -0.120503 1.278028 -0.696543

9 6 0 1.035064 1.983733 -1.520608

10 6 0 2.324455 1.199090 -1.198697

11 6 0 1.770283 -0.244731 -1.084020

12 7 0 2.522458 -1.202139 -0.326572

13 6 0 1.963933 -1.382159 0.910585

14 6 0 -2.983628 -0.455740 -0.065382

15 6 0 -3.525598 0.621515 -0.971686

16 6 0 -3.244691 1.917951 -0.198826

17 6 0 -3.229948 -1.906630 -0.375838

18 6 0 -4.704193 -2.301802 -0.230493

19 6 0 -2.730663 -2.259767 -1.781667

20 8 0 -1.401491 -1.833104 -2.034825

21 8 0 2.502934 -2.005779 1.832233

22 6 0 3.789371 -1.793618 -0.685644

23 6 0 4.975821 -0.900942 -0.309584

24 8 0 4.761527 -0.261031 0.944208

25 1 0 -0.900347 0.996170 -1.414362

26 1 0 1.518769 -0.621437 -2.084504

27 8 0 2.851026 1.649334 0.025265

28 6 0 1.187639 3.486349 -1.346165

29 6 0 -2.517030 2.258751 2.208466

30 1 0 0.089051 0.461532 2.680431

31 1 0 0.288504 -1.257473 2.923067

32 1 0 -2.087016 -0.460852 3.014136

33 1 0 -1.805862 -1.791924 1.912567

34 1 0 -0.087276 2.111557 1.290590

35 1 0 -0.893733 3.123276 0.116998

36 1 0 0.826802 1.790063 -2.580723

37 1 0 3.062467 1.292103 -2.010320

38 1 0 -3.019840 0.596016 -1.947685

39 1 0 -4.594495 0.491020 -1.183992

40 1 0 -2.930047 2.740095 -0.850776

41 1 0 -4.161175 2.246215 0.304857

42 1 0 -2.654862 -2.524778 0.326672

43 1 0 -4.846836 -3.362678 -0.465627

44 1 0 -5.342750 -1.721171 -0.905073

45 1 0 -5.052168 -2.131596 0.792764

46 1 0 -2.823979 -3.347985 -1.924634

47 1 0 -3.360120 -1.777216 -2.538934

48 1 0 -0.912352 -1.875854 -1.198184

49 1 0 3.857912 -2.739049 -0.139055

50 1 0 3.804342 -2.020309 -1.756195

51 1 0 5.893997 -1.502681 -0.296053

52 1 0 5.116201 -0.096881 -1.038826

53 1 0 4.308741 -0.902353 1.526646

54 1 0 3.503418 0.996712 0.365563

55 1 0 0.278566 4.016142 -1.649170

56 1 0 2.008213 3.854773 -1.972334

57 1 0 1.413163 3.748852 -0.309629

58 1 0 -1.789302 2.013519 2.990698

59 1 0 -3.508883 1.953266 2.560530

60 1 0 -2.521778 3.349595 2.093981

-------------------------------------------------------------------------------------------------------------

C5

Standard orientation:

-------------------------------------------------------------------------------------------------------------

Center Atomic Atomic Coordinates (Angstroms)

Number Number Type X Y Z

-------------------------------------------------------------------------------------------------------------

1 6 0 0.587146 0.352980 -0.217165

2 6 0 0.803029 -0.428927 0.858597

3 6 0 -0.002592 -0.661660 2.095652

4 6 0 -1.459747 -1.104171 1.869028

5 6 0 -2.258126 -0.274661 0.903780

6 6 0 -2.376359 1.246363 1.040002

7 6 0 -1.081271 2.020787 0.662611

8 6 0 -0.351927 1.471866 -0.566708

9 6 0 0.599922 2.443624 -1.372387

10 6 0 2.032056 1.939007 -1.115479

11 6 0 1.816493 0.411590 -1.066511

12 7 0 2.782622 -0.391708 -0.350412

13 6 0 2.176116 -0.981860 0.732715

14 6 0 -2.990145 -0.754920 -0.123779

15 6 0 -3.695583 0.351698 -0.871072

16 6 0 -3.517458 1.577011 0.035249

17 6 0 -3.120585 -2.189671 -0.574304

18 6 0 -4.558757 -2.566111 -0.942664

19 6 0 -2.183941 -2.471263 -1.758129

20 8 0 -0.815847 -2.370977 -1.413908

21 8 0 2.702509 -1.799714 1.496120

22 6 0 3.984352 -0.905152 -1.005270

23 6 0 5.229508 -0.945711 -0.121471

24 8 0 5.281270 -2.052138 0.745170

25 1 0 -1.110063 1.112823 -1.274922

26 1 0 1.661373 0.026313 -2.083660

27 8 0 2.469000 2.420025 0.138023

28 6 0 0.430227 3.934259 -1.126312

29 6 0 -2.777662 1.661440 2.460825

30 1 0 0.013497 0.250936 2.704882

31 1 0 0.507795 -1.426906 2.690177

32 1 0 -1.953628 -1.096567 2.851079

33 1 0 -1.452228 -2.146279 1.536987

34 1 0 -0.386727 2.052567 1.509546

35 1 0 -1.378227 3.060074 0.482805

36 1 0 0.406759 2.256782 -2.436633

37 1 0 2.721495 2.243658 -1.916029

38 1 0 -3.255535 0.499274 -1.868960

39 1 0 -4.753597 0.123683 -1.045127

40 1 0 -3.313136 2.492933 -0.528921

41 1 0 -4.443847 1.751225 0.594094

42 1 0 -2.798336 -2.853489 0.236820

43 1 0 -4.630177 -3.640283 -1.144056

44 1 0 -4.899353 -2.036528 -1.839271

45 1 0 -5.251551 -2.328206 -0.129070

46 1 0 -2.336747 -3.498447 -2.107303

47 1 0 -2.435815 -1.800004 -2.596046

48 1 0 -0.697215 -1.527171 -0.944216

49 1 0 3.799658 -1.911199 -1.405308

50 1 0 4.176172 -0.240596 -1.852624

51 1 0 6.101732 -1.017461 -0.780550

52 1 0 5.309991 0.011782 0.420949

53 1 0 4.403508 -2.076259 1.189661

54 1 0 3.189985 1.838978 0.430194

55 1 0 -0.589728 4.259242 -1.356562

56 1 0 1.110837 4.500688 -1.771739

57 1 0 0.652792 4.200045 -0.089786

58 1 0 -2.000405 1.419595 3.194635

59 1 0 -3.702319 1.160417 2.768266

60 1 0 -2.948966 2.743654 2.507092

-------------------------------------------------------------------------------------------------------------

**Coordinates of computation** (2*S*,3*R*,4*S*,5*R*,11*R*,15*R*)-**3**

Standard orientation:

-------------------------------------------------------------------------------------------------------------

Center Atomic Atomic Coordinates (Angstroms)

Number Number Type X Y Z

-------------------------------------------------------------------------------------------------------------

1 6 0 -0.658740 0.499821 -0.114080

2 6 0 -0.513210 -0.579431 -0.900603

3 6 0 0.554015 -1.129038 -1.786290

4 6 0 1.804785 -0.281788 -2.023268

5 6 0 2.483436 0.233415 -0.783651

6 6 0 2.471241 1.723682 -0.472406

7 6 0 1.090748 2.390388 -0.614492

8 6 0 -0.056168 1.833711 0.223567

9 6 0 3.246522 -0.452127 0.084323

10 6 0 3.837754 0.464238 1.131544

11 6 0 3.039907 1.766659 0.964524

12 6 0 -2.003582 0.447550 0.574819

13 7 0 -2.650290 -0.710964 0.009596

14 6 0 -1.784460 -1.369364 -0.809496

15 6 0 -1.330629 2.770161 0.316140

16 6 0 -2.572720 1.835142 0.264330

17 8 0 -2.019162 -2.412933 -1.429662

18 8 0 -1.934810 0.402263 1.984798

19 6 0 3.597789 -1.912930 0.026548

20 6 0 5.100914 -2.126800 -0.200873

21 6 0 3.171603 -2.662017 1.295742

22 6 0 3.431914 2.441263 -1.438606

23 6 0 -1.447436 3.859370 -0.744073

24 1 0 0.299690 1.735472 1.259297

25 8 0 -3.629860 2.215264 1.113145

26 6 0 -3.961311 -1.171442 0.446506

27 6 0 -4.925772 -1.512930 -0.687171

28 8 0 -4.705094 -2.782291 -1.256657

29 8 0 1.793578 -2.541082 1.607801

30 6 0 -1.264335 -0.723182 2.540348

31 1 0 0.100021 -1.387902 -2.751649

32 1 0 0.848988 -2.096110 -1.355344

33 1 0 1.538309 0.565970 -2.665631

34 1 0 2.499658 -0.892624 -2.613216

35 1 0 0.801990 2.395084 -1.673680

36 1 0 1.218792 3.444976 -0.338022

37 1 0 3.764570 0.061802 2.149561

38 1 0 4.910485 0.618337 0.942974

39 1 0 2.224765 1.781882 1.696071

40 1 0 3.642908 2.664117 1.139859

41 1 0 -1.328071 3.251530 1.300872

42 1 0 -2.978607 1.818276 -0.753137

43 1 0 3.065681 -2.370827 -0.819310

44 1 0 5.334470 -3.194106 -0.285732

45 1 0 5.690763 -1.720937 0.628784

46 1 0 5.430570 -1.633109 -1.120179

47 1 0 3.459640 -3.719916 1.200747

48 1 0 3.707377 -2.258011 2.161464

49 1 0 3.447888 3.520282 -1.241584

50 1 0 4.452579 2.059120 -1.326938

51 1 0 3.131774 2.298047 -2.483485

52 1 0 -0.650921 4.605255 -0.669383

53 1 0 -2.402085 4.382813 -0.618677

54 1 0 -1.428586 3.441364 -1.757053

55 1 0 -3.337540 1.972051 2.008911

56 1 0 -3.860473 -2.050519 1.097062

57 1 0 -4.378167 -0.357531 1.045099

58 1 0 -5.939810 -1.524657 -0.272463

59 1 0 -4.888246 -0.707137 -1.438945

60 1 0 -3.743023 -2.817488 -1.456532

61 1 0 1.295321 -3.000370 0.916790

62 1 0 -1.234183 -0.553216 3.617525

63 1 0 -0.243410 -0.820127 2.154684

64 1 0 -1.811892 -1.650900 2.338377

----------------------------------------------------------------------------------------------------------------

4. Antimicrobial assay and Cytotoxicity assay of 1−3

**Antimicrobial assay**

Compounds were tested for the antimicrobial activity using a paper disk-diffusion assay.^1, 2^ Seed cultures of two bacteria (*S. aureus* 209P, *E. coli* ATCC0111) and two fungi (*C. albicans* FIM709, *A. niger* R330) were prepared by incubating the organism for 12 h at 32 °C (fungi) or 37 °C (bacteria). Aliquots of the overnight cultures (80 μL) were spread onto the surfaces of nutrient agar (bacteria) or Sabouraud’s dextrose agar (fungi). Sterile filter disks (6 mm diameter) infused with 6 μL of test solution (in DMSO), positive control and vehicle only (DMSO) were added to the plates. The plates were left upright for 30 min at room temperature before being placed in an incubator at 32 °C (fungi) or 37 °C (bacteria) for 12 hours, and then the diameter of the zone of growth inhibition for each disk was recorded. The continuous twofold dilution methods were used to evaluate the minimal inhibitory concentrations. The MICs were defined as the lowest concentration at which no microbial growth could be observed.

Table S4.Antimicrobial activity of **1**−**3** (MIC, µg/ml)

|  | Bacteria | |  | Fungi | |
| --- | --- | --- | --- | --- | --- |
| no. | *S. aureus* | *E. coli* |  | *C. albicans* | *A. niger* |
| **1** | >1000 | >1000 |  | >1000 | >1000 |
| **2** | >1000 | >1000 |  | >1000 | >1000 |
| **3** | >1000 | >1000 |  | >1000 | >1000 |
| Tobramycin^a^ | <16 | <16 |  | nt | nt |
| Cycloheximide^b^ | nt | nt |  | <16 | <16 |

^a^Positive control (bacteria). ^b^Positive control (fungi).

nt: not tested

**Cytotoxicity assay**

Five human cancer cell lines, human myeloid leukemia HL-60, hepatocellular carcinoma SMMC-7721, lung cancer A-549, breast cancer MCF-7, and colon cancer SW480, were used in the cytotoxicity assay. All the cells were cultured in DMEM medium (Hyclone, USA), supplemented with 10% fetal bovine serum (Hyclone, USA), in 5% CO_2_ at 37 °C. The cytotoxicity assay was performed according to the MTT (3-(4,5-dimethylthiazol-2-yl)-2,5-diphenyl tetrazolium bromide) method in 96-well microplates^3, 4^ with cisplatin and paclitaxel (Sigma, USA) as the positive controls. Cell viability after 48 h treatment was detected and cell growth curve was graphed. The IC_50_ values were calculated by Reed and Muench’s method.^5, 6^

Table S5. Cytotoxicity of **1**−**3** against human tumor cell lines (IC_50_, µm)

| no. | HL-60 | SMMC-7721 | A-549 | MCF-7 | SW480 |
| --- | --- | --- | --- | --- | --- |
| **1** | >40 | >40 | >40 | >40 | >40 |
| **2** | >40 | >40 | >40 | >40 | >40 |
| **3** | >40 | >40 | >40 | >40 | >40 |
| cisplatin | 1.93 | 10.21 | 6.59 | 8.20 | 12.16 |
| paclitaxel | <0.008 | <0.008 | <0.008 | <0.008 | <0.008 |

**References**

1. Groblacher, B., Maier, V., Kunert, O., & Bucar, F. *J Nat Prod* **75**, 1393–1399 (2012).
2. Shen, C. C., Syu, W. J., Li, S. Y., Lin, C. H., Lee, G. H., & Sun, C. M. *J Nat Prod* **65**, 1857–1862 (2002).
3. Mosmman, T. *J Immunol Methods* **65**, 55–63 (1983).
4. Alley, M. C., Scudiero, D. A., Monks, A., Hursey, M. L., Czerwinski, M. J., & Fine, D. L. *Cancer Res* **48**, 589–601 (1988).
5. Reed, L. J., Muench, H. *Am J Hyg* **27**, 493–497 (1938).
6. Chen, G. D., *et al*. *J Nat Prod* **76**, 702–709 (2013).

5. The spectra of 1−3

**UV spectrum of 1**

**IR spectrum of 1**

**
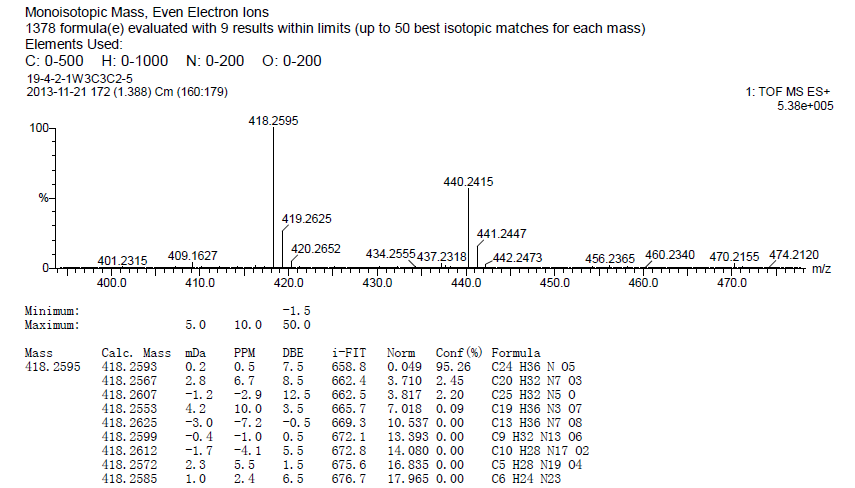
**

**HRESIMS spectrum of 1**

**^1^H NMR spectrum of 1 in CD_3_OD (400 MHz)**

**^13^C NMR spectrum of 1 in CD_3_OD (100MHz)**

**DEPT-135 spectrum of 1 in CD_3_OD**

**^1^H-^1^H COSY spectrum of 1 in CD_3_OD**

**HSQC spectrum of 1 in CD_3_OD**


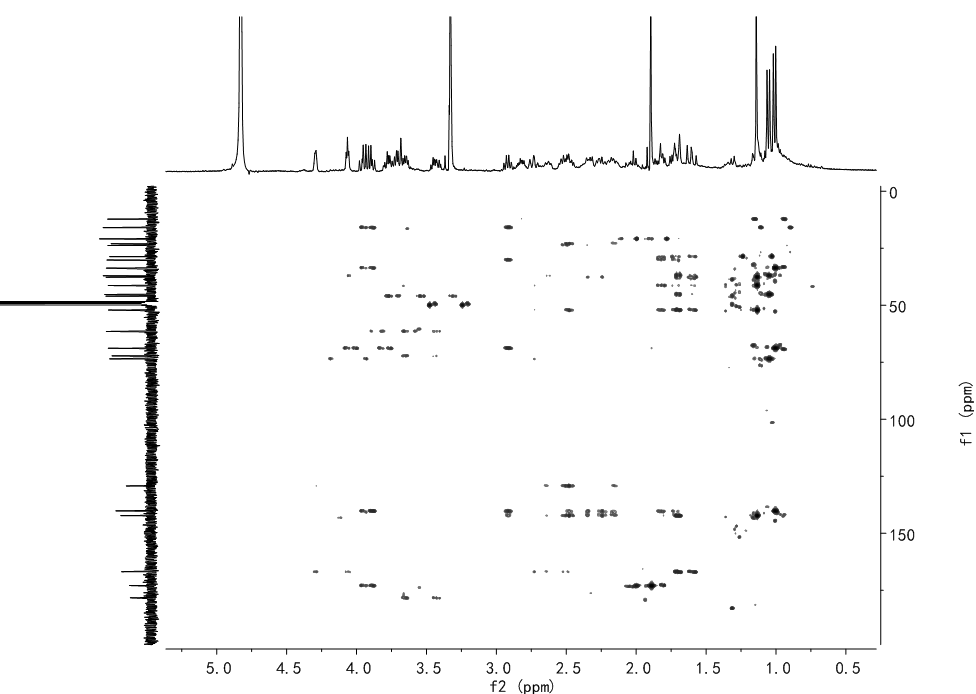


**HMBC spectrum of 1 in CD_3_OD**

**
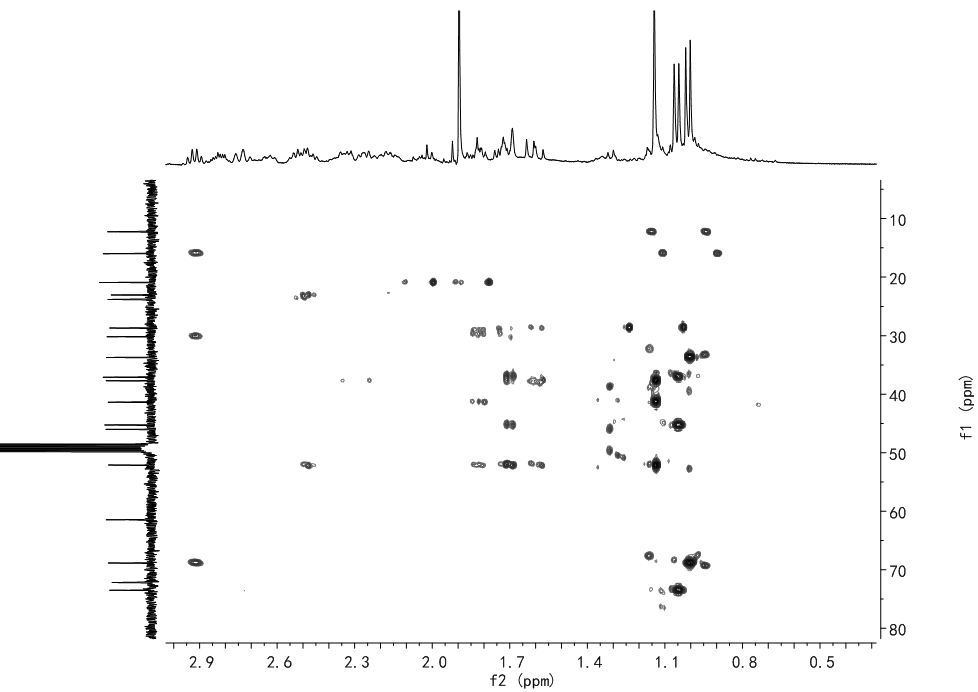
**

**Magnified HMBC spectrum of 1 in CD_3_OD**

**
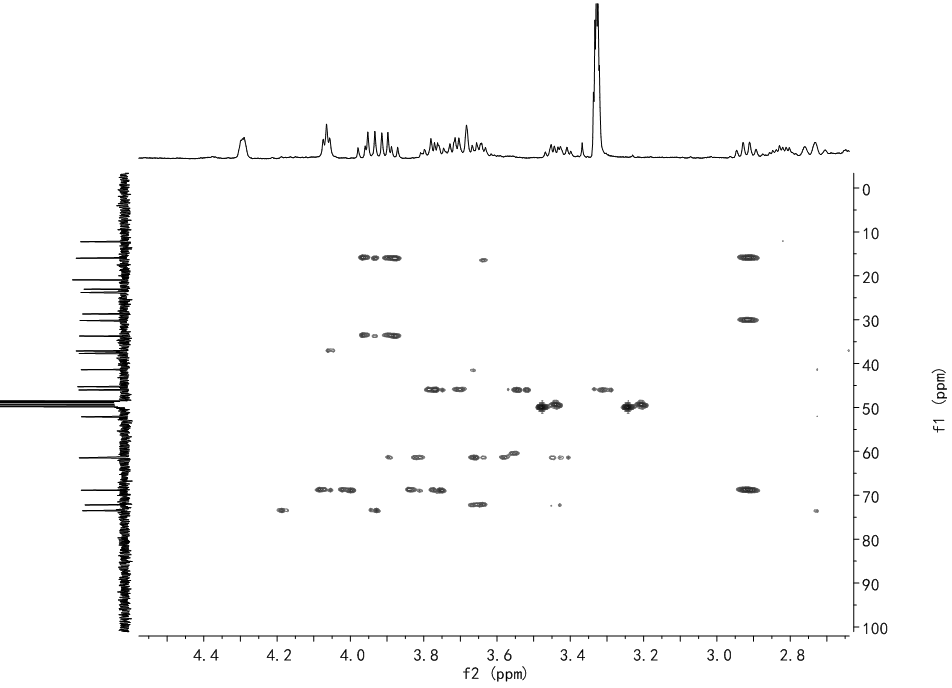
Magnified HMBC spectrum of 1 in CD_3_OD**

**
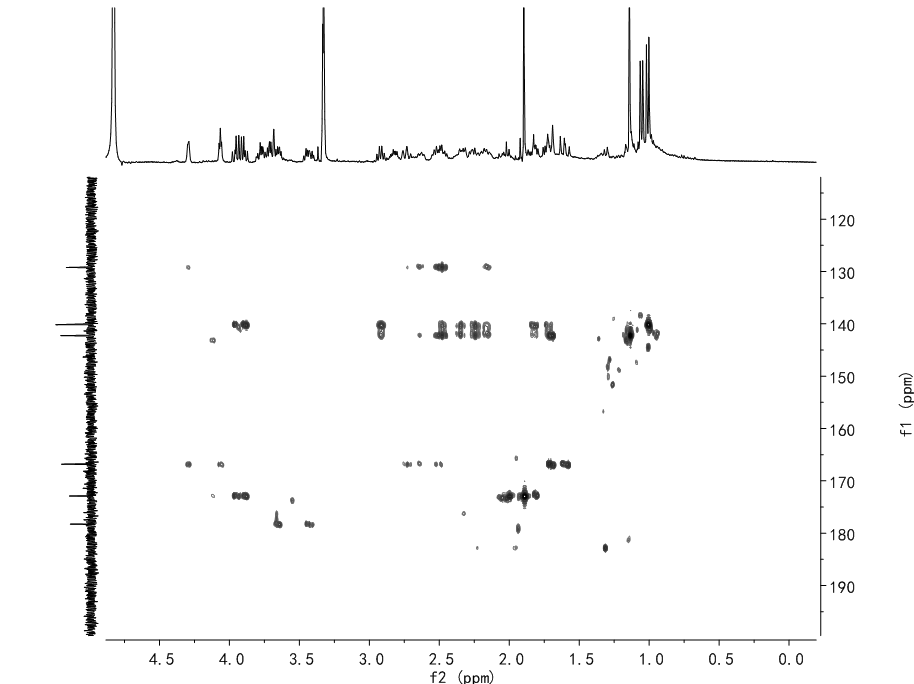
Magnified HMBC spectrum of 1 in CD_3_OD**

**ROESY spectrum of 1 in CD_3_OD**

**UV spectrum of 2**

**IR spectrum of 2**


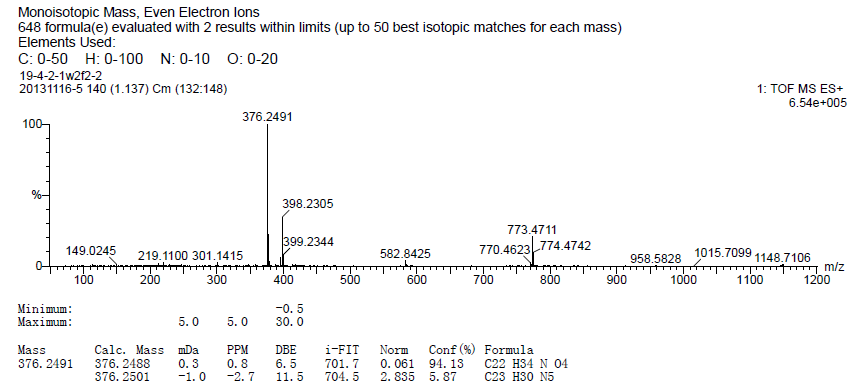


**HRESIMS spectrum of 2**

**^1^H NMR spectrum of 2 in CD_3_OD (400 MHz)**

**^13^C NMR spectrum of 2 in CD_3_OD (100 MHz)**

**DEPT-135 spectrum of 2 in CD_3_OD**

**^1^H-^1^H COSY spectrum of 2 in CD_3_OD**

**HSQC spectrum of 2 in CD_3_OD**


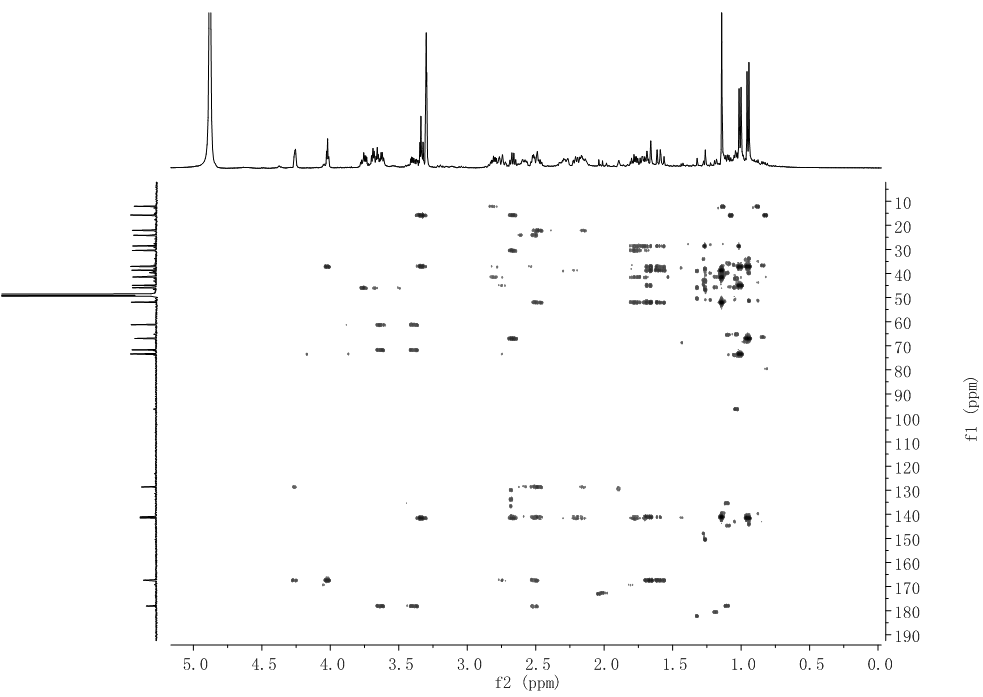


**HMBC spectrum of 2 in CD_3_OD**

**
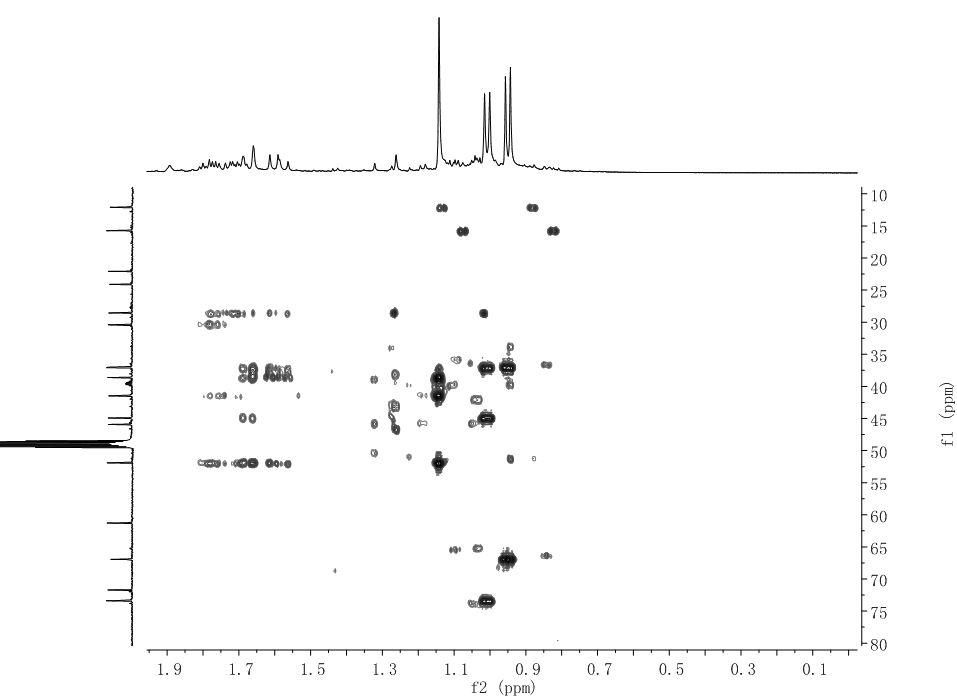
Magnified HMBC spectrum of 2 in CD_3_OD**

**
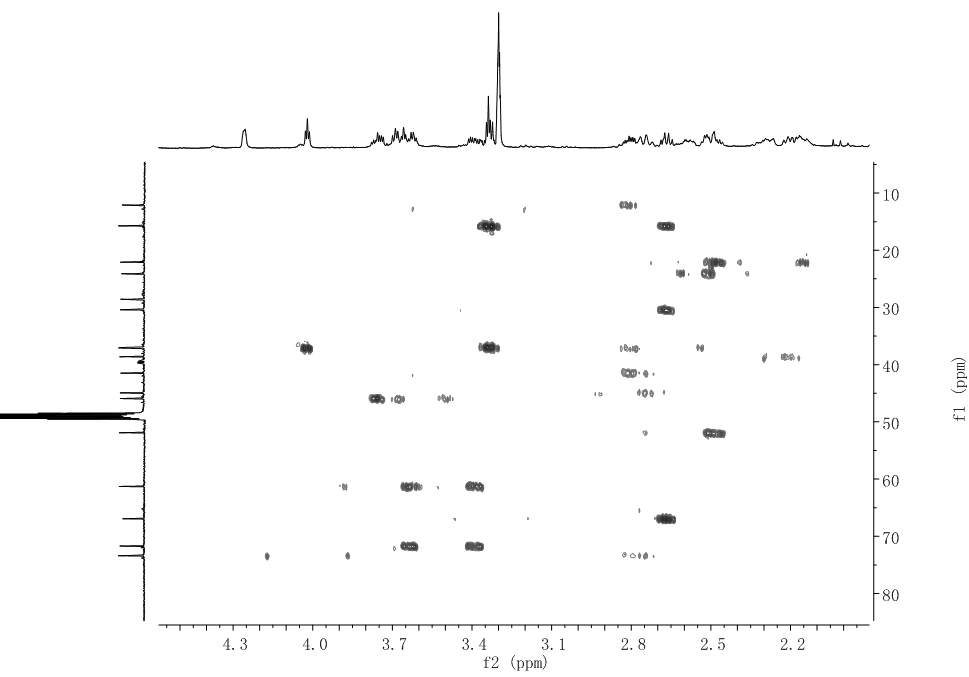
Magnified HMBC spectrum of 2 in CD_3_OD**

**ROESY spectrum of 2 in CD_3_OD**

**UV spectrum of 3**

**IR spectrum of 3**


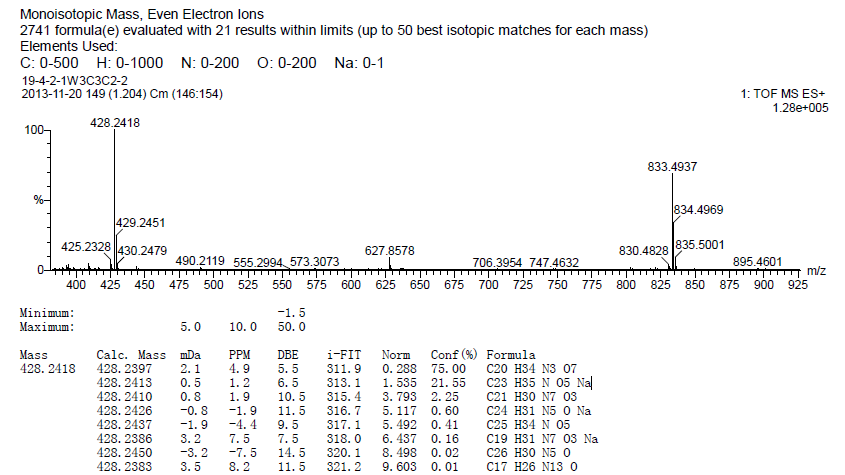


**HRESIMS spectrum of 3**

**^1^H NMR spectrum of 3 in CD_3_OD (400 MHz)**

**^13^C NMR spectrum of 3 in CD_3_OD (100 MHz)**

**DEPT-135 spectrum of 3 in CD_3_OD**

**^1^H-^1^H COSY spectrum of 3 in CD_3_OD**

**HSQC spectrum of 3 in CD_3_OD**

**HMBC spectrum of 3 in CD_3_OD**

**
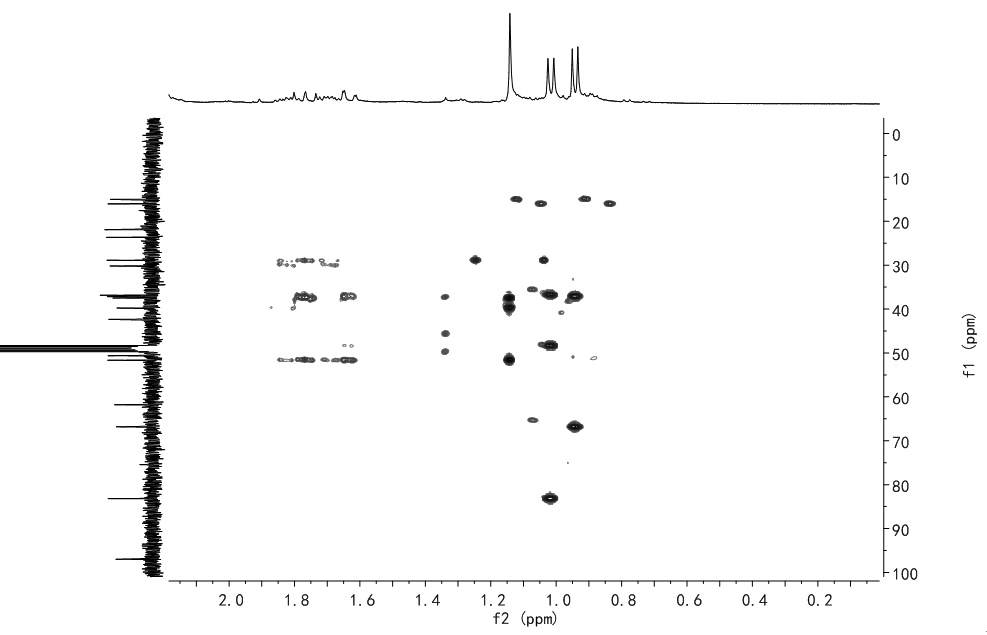
Magnified HMBC spectrum of 3 in CD_3_OD**

**
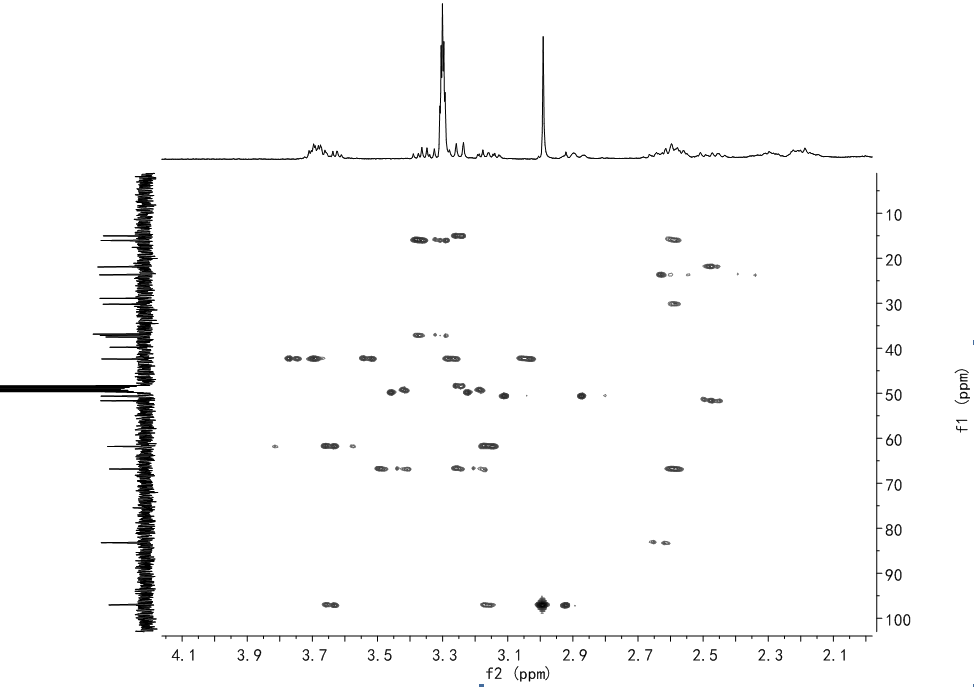
**

**Magnified HMBC spectrum of 3 in CD_3_OD**

**
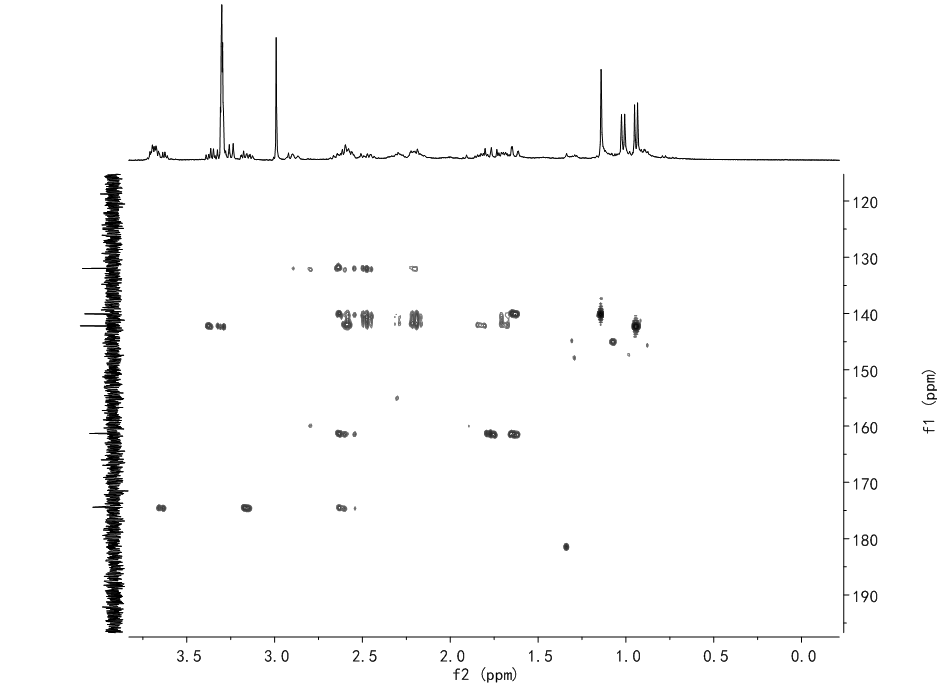
Magnified HMBC spectrum of 3 in CD_3_OD**

**ROESY spectrum of 3 in CD_3_OD**
